# Unlocking the Potential of Sea Fennel, an Emerging Food Crop: Physicochemical, Microbial, and Aromatic Traits Shaped by Fermentation and Pickling

**DOI:** 10.3390/foods15081450

**Published:** 2026-04-21

**Authors:** Maryem Kraouia, Antonietta Maoloni, Aizhan Ashim, Benedetta Fanesi, Lama Ismaiel, Deborah Pacetti, Giorgia Rampanti, Federica Cardinali, Vesna Milanovic, Cristiana Garofalo, Andrea Osimani, Lucia Aquilanti

**Affiliations:** Dipartimento di Scienze Agrarie, Alimentari e Ambientali (D3A), Università Politecnica delle Marche, 60131 Ancona, Italy; m.kraouia@pm.univpm.it (M.K.); a.maoloni@univpm.it (A.M.); a.ashim@pm.univpm.it (A.A.); b.fanesi@pm.univpm.it (B.F.); l.ismaiel@staff.univpm.it (L.I.); d.pacetti@staff.univpm.it (D.P.); f.cardinali@univpm.it (F.C.); v.milanovic@univpm.it (V.M.); c.garofalo@univpm.it (C.G.); a.osimani@univpm.it (A.O.); l.aquilanti@staff.univpm.it (L.A.)

**Keywords:** *Crithmum maritimum* L., lactic acid fermentation, pickling, mild pasteurization, sensory analysis, volatile organic compounds

## Abstract

Sea fennel (*Crithmum maritimum* L.) is an emerging crop valued for its nutritional and sensory properties and has been reported to exert health-promoting effects, including antioxidant, anti-inflammatory, antimicrobial, and cardioprotective activities, as well as potential benefits for gut health and metabolic regulation. Building on these features, the present study aimed to unlock the potential of sea fennel to produce novel pickles. Two independent batches were prepared using young leaves and stems of sea fennel fermented in brine. After fermentation, salt concentration was standardized in all prototypes, and two types of vinegar (apple and wine) were added at four acetic acid levels (0.05%, 0.2%, 0.5%, and 0.7%). All prototypes were subsequently subjected to mild pasteurization. During fermentation, physicochemical and microbiological parameters were monitored, while after pasteurization additional physicochemical, microbiological, volatile organic compound (VOCs), and sensory analyses were performed during storage. In both batches and across all prototypes, fermentation resulted in a significant pH decrease, dominance of lactic acid bacteria, inhibition of Enterobacteriaceae, and a gradual increase in yeasts. Following vinegar addition and pasteurization, pH, titratable acidity, and salt content remained stable over six months of storage in most prototypes, particularly those with 0.2% acetic acid. Pasteurization effectively inactivated lactic acid bacteria and Enterobacteriaceae in all prototypes, whereas yeasts and mesophilic bacteria persisted in low-acidity samples (0.05%). Therefore, the 0.05% acidity samples were later excluded due to mid-stage microbial spoilage. Batch-dependent differences were observed in color and sensory attributes, with batch 2 showing higher overall stability mainly in acidic flavor and aroma, particularly in prototypes with 0.2% acidity. VOCs analysis revealed profiles primarily driven by batch variation, with secondary modulation by vinegar type: sesquiterpenes remained stable, while γ-terpinene, limonene, and *p*-cymene were the dominant compounds, with greater stability observed in batch 2. Overall, the combined use of lactic acid fermentation, vinegar pickling, and mild pasteurization represents a promising strategy for preserving sea fennel and supports its potential as a vegetable crop.

## 1. Introduction

Sea fennel (*Crithmum maritimum* L.), also known as rock samphire or Saint Peter’s herb, is a perennial halophyte naturally growing along maritime cliffs and sandy shores of the Mediterranean and Atlantic coasts [1,2]. It is increasingly recognized as an emerging food crop due to its high salt tolerance, distinctive sensory traits, and richness in bioactive compounds [3,4]. Its succulent leaves and young shoots are valued for their crisp texture and aromatic, kerosene-like flavor, and are traditionally consumed fresh, pickled, or used as a seasoning [5]. Nutritionally, sea fennel represents a valuable source of vitamins, carotenoids, polyphenols, essential ω-3 and ω-6 fatty acids, and volatile oils, with reported antioxidant, antimicrobial, and anti-inflammatory properties [6,7,8]. These features highlight its potential both as a nutritionally valuable crop and as a promising ingredient for innovative preserves production.

Pickling is among the oldest and most widespread food preservation techniques, historically practiced for more than three millennia across diverse cultures, including ancient Egyptian, Chinese, and Indian civilizations [9,10]. It involves the preservation of foods in brine or vinegar and has been extensively applied to vegetables such as cabbages, cucumbers, onions, radishes, carrots, and legumes [11,12,13]. In recent years, there has been an increasing scientific interest in fermented and acidified vegetables due to their potential to combine preservation stability with sensory quality and bioactive properties [14].

Vegetable pickles can be obtained either through spontaneous fermentation, in which naturally occurring lactic acid bacteria convert sugars into lactic acid, or through direct acidification with vinegar, with or without the addition of salt. Fermentation contributes to microbial safety, flavor development, and nutritional enhancement through the synthesis of bioactive compounds, bacteriocins, and exopolysaccharides [15,16,17].

Beyond serving as an alternative to direct acidification, fermentation may also act as a preliminary stabilization step prior to vinegar addition, forming a two-step preservation strategy that integrates both processes [18]. This combined approach is consistent with traditional and industrial preservation practices, in which vegetables are stabilized by fermentation, acidification, or their combination, often coupled with pasteurization or refrigeration to extend shelf life.

Pasteurization is commonly applied to ensure product stability during storage by inactivating vegetative microorganisms and enzymes [19]. Fermentation relies on the activity of lactic acid bacteria to produce nutritious foods that can remain stable for up to one year without refrigeration [20], mainly due to the accumulation of organic acids and antimicrobial compounds such as lactic and acetic acids [15]. Moreover, lactic acid bacteria contribute to the production of minimally processed foods with enhanced safety, reducing the need for chemical preservatives [21,22,23]. The subsequent addition of vinegar further improves product stability and supports the preservation of natural bioactive compounds and antioxidant activity [24]. Therefore, combining fermentation with vinegar pickling represents an effective strategy for producing stable, high-quality vegetables with extended shelf life, as vinegar contributes to maintaining an acidic environment that inhibits microbial spoilage while enhancing organic acids, polyphenols, and other functional compounds with potential health benefits [25,26,27].

Given the growing interest in halophytes as climate-resilient crops, sea fennel represents an excellent candidate for the development of novel pickled vegetable products. Previous studies have investigated sea fennel as a fermented or acidified vegetable product, including spontaneous or starter-driven fermentation in brine [7] and vinegar-preserved pickles [28]. However, an integrated approach combining controlled lactic acid fermentation, vinegar addition, and pasteurization has not yet been fully explored. Building on these premises and on the previous findings by Maoloni et al. [7], demonstrating that a selected multi-strain lactic acid bacteria starter effectively controls sea fennel fermentation in brine, ensuring reproducible acidification, microbiological stability, and product quality of sea fennel preserves, the present study aims to investigate the combined effects of fermentation, vinegar addition, and pasteurization on the physicochemical, microbiological, sensory, and volatile characteristics of sea fennel pickles. In more detail, the experimental plan consisted of sea fennel pickle production in two independent batches using this combined preservation approach. In each batch, young leaves and stems of sea fennel were fermented in brine using a multi-strain starter culture of lactic acid bacteria. Three biological replicates of fermented sea fennel were prepared for each independent batch and monitored throughout fermentation (20 days) by physicochemical and microbiological analyses to assess microbial dynamics, pH, and acidity. After fermentation, prototypes were subjected to salt stabilization for five months. Subsequently, fermented sea fennel samples were desalted, pooled, and processed to obtain four prototypes for each type of vinegar (apple and wine), each at four target acetic acid concentrations (0.05%, 0.2%, 0.5%, and 0.7%), resulting in eight distinct vinegar–acidity combinations per batch. All final pickle prototypes were then subjected to mild pasteurization and monitored over six months of storage to evaluate physicochemical, microbiological, volatile, and sensory characteristics.

## 2. Materials and Methods

### 2.1. Experimental Design

**Figure 1:**
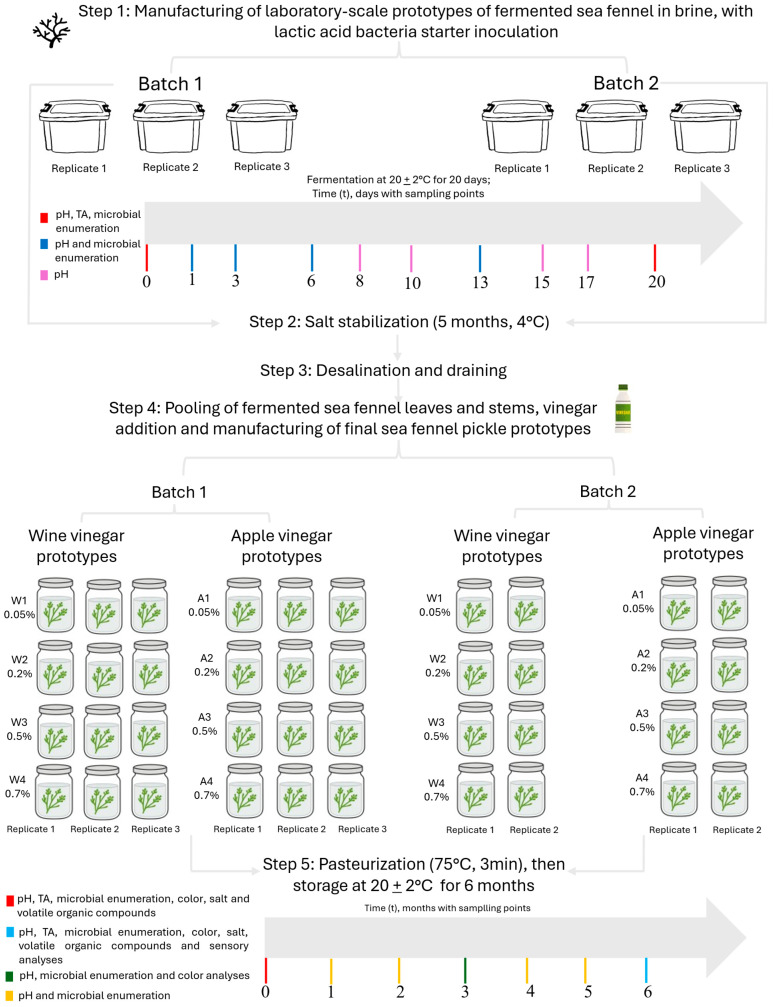
Experimental plan for the manufacturing of laboratory scale prototypes of sea fennel pickles produced by the combined approach of fermentation and vinegar pickling. Two independent batches were prepared, first batch (B1), and the second one (B2). In each batch, the following steps were performed: step 1: manufacturing of laboratory-scale prototypes of fermented sea fennel in brine; step 2: salt stabilization; step 3: desalination and draining; step 4: vinegar addition to the fermented sea fennel leaves and stems and manufacturing of final pickle prototypes, step 5: pasteurization then 6-months storage of all the laboratory-scale pickle prototypes.

The research was conducted through two main pickling steps: (i) fermentation of fresh young sea fennel leaves and stems in brine using a starter culture previously formulated by Maoloni et al. [7]; and (ii) addition of either apple or wine vinegar to the fermented sea fennel. The overall experimental design is illustrated in Figure 1, while detailed information of each step is provided in the following sections.

### 2.2. Starter Culture

A multi-strain starter culture of lactic acid bacteria was used to guide the fermentation process. The same starter has previously been employed to produce fermented sea fennel preserves in brine [7], fermented table olives with sea fennel [29], and sea fennel–enriched kimchi [30]. The starter culture consisted of four strains: *Lactiplantibacillus plantarum* PB257, *Leuconostoc pseudomesenteroides* PB288, *Pediococcus pentosaceus* FF78, and *Weissella confusa* PB321. All strains belong to the Culture Collection of the Department of Agricultural, Food and Environmental Sciences (D3A), Università Politecnica delle Marche, and were stored at −80 °C in de Man, Rogosa and Sharpe (MRS) broth (VWR International, Radnor, PA, USA) supplemented with glycerol (final ratio 3:2, broth:glycerol). The starter culture was prepared as described by Maoloni et al. [7]. Briefly, prior to use, strains were subcultured in MRS broth at 30 °C for 24 h. Cultures were then centrifuged at 4000 rpm for 10 min, and the obtained cell pellets were washed twice with sterile 0.85% (w v^−1^) NaCl solution. The washed cells were subsequently resuspended in sterile saline solution, and cell density was estimated by measuring optical density at 600 nm using a UV–Vis Shimadzu UV-1800 spectrophotometer (Shimadzu Corporation, Kyoto, Japan). The microbial concentration was further verified by viable plate counts on MRS agar (VWR) incubated at 30 °C for 48–72 h.

### 2.3. Sea Fennel Crop Supply and Pretreatment

Laboratory-scale prototypes of fermented sea fennel pickles were produced using organic fresh sea fennel leaves and stems obtained from two independent harvests carried out in November 2022 and July 2023 at a local farm producing organic sea fennel (Rinci S.r.l., Castelfidardo, Ancona, Italy). For each harvest, 5 kg of fresh sea fennel leaves and stems were collected immediately after harvesting, transported to the laboratory in sterile plastic bags, thoroughly washed under tap water, blanched at 95 °C for 30 s, and drained for 10 min on blotting paper at room temperature prior to processing.

### 2.4. Pickling of Sea Fennel—Phase One: Characterization of Fermentation Process

**Figure 2:**
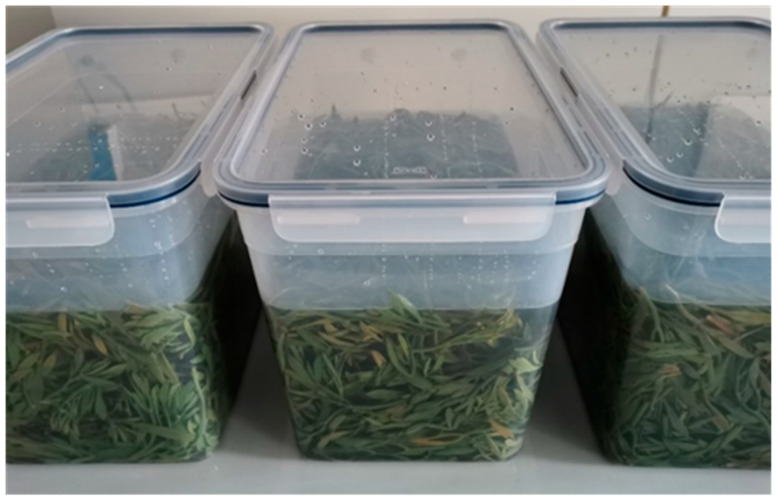
Fermentation of sea fennel leaves and stems in brine in plastic boxes.

Fermentation was carried out in food-grade plastic containers, previously cleaned with alcohol and hermetically sealed with lids (Figure 2). Two independent batches were produced, referred to as batch 1 (B1) and batch 2 (B2) to evaluate the inter-batch reproducibility, each consisting of three biological replicates (hereafter referred to as containers). For each container, 1795 g of blanched sea fennel were combined with 5385 mL of brine composed of a 7% (w v^−1^) NaCl solution, sterilized by autoclaving, and supplemented with 1% (w v^−1^) fructose previously sterilized by filtration. To ensure a standardized initial balance, the four strains of the starter culture were inoculated individually into the brine in an equal 1:1:1:1 ratio to reach a final load of ∼7 Log CFU mL^−1^ in brine. Fermentation was conducted at room temperature (20 ± 2 °C) for 20 days.

#### 2.4.1. pH Measurement

Aliquots (1 mL) of brine were aseptically collected from each container immediately after inoculation and at regular intervals throughout fermentation, corresponding to days 1, 3, 6, 8, 10, 13, 15, 17, and 20. The pH was measured using a pH meter (model 300; Hanna Instruments, Padova, Italy). Results are expressed as the mean ± standard deviation of three biological replicates for each batch (B1 and B2).

#### 2.4.2. Titratable Acidity (TA) Measurement

Titratable acidity (TA) was determined on days 0 and 20 of fermentation, according to the method described by Rampanti at al. [31]. Briefly, 10 mL of each brine sample was homogenized with 90 mL of distilled water and titrated with a 0.1 N NaOH solution until reaching a pH of 8.3. TA was expressed as a percentage of lactic acid equivalents using the following formula:

$$TA=\frac{Volume\;of\;titrant\times N\times 90}{Weight\;of\;sample\times 1000}\times 100$$

where *N* is the normality of the titrant, and 90 represents the equivalent molecular weight of lactic acid. Results are reported as mean ± standard deviation of three biological replicates in B1 and B2.

#### 2.4.3. Microbial Counts

Microbiological analyses were performed on brine samples (1 mL) aseptically collected immediately after production (day 0) and at selected time points during fermentation (days 1, 3, 6, 13, and 20). Each sample was serially tenfold diluted in sterile 0.1% (w v^−1^) peptone water. Appropriate dilutions were plated on selective media for the enumeration of the following microbial groups: (i) mesophilic aerobic bacteria and spore-forming bacteria on Plate Count Agar (PCA; VWR International Srl, Milan, Italy), incubated at 30 °C for 48 h; (ii) presumptive mesophilic lactobacilli on de Man, Rogosa and Sharpe (MRS) agar (VWR) supplemented with cycloheximide (100 mg L^−1^; VWR) to inhibit yeast growth, incubated at 30 °C for 48 h; (iii) yeasts and molds on Rose Bengal Chloramphenicol Agar (RB; VWR), incubated at 25 °C for 72 h; and (iv) Enterobacteriaceae on Violet Red Bile Glucose Agar (VRBGA; VWR), incubated at 37 °C for 24 h.

For the selective enumeration of spore-forming bacteria, brine samples were subjected to heat treatment at 80 °C for 10 min to inactivate vegetative cells, followed by rapid cooling in ice water prior to plating. Viable counts were expressed as mean log colony-forming units (CFU) per mL of brine ± standard deviation of three biological replicates for each batch (B1 and B2).

### 2.5. Stabilization

After 20 days of fermentation, the brine in all containers was adjusted to a final NaCl concentration of 8% (w v^−1^) and stored at 4 °C for five months prior to vinegar addition. The pH was monitored at the beginning and at the end of the storage period as described in Section 2.4.1. and remained stable at approximately 3.6 throughout storage.

### 2.6. Pickling of Sea Fennel—Phase 2: Desalinization, Distribution in Glass Jars and Vinegar Addition

At the end of the stabilization period in each batch, fermented sea fennel leaves and stems were drained using a stainless-steel strainer, rinsed under tap water, and immersed in mineral water at a ratio of 1:3 (w v^−1^) for approximately 60 h at room temperature (20 ± 2 °C) to allow desalination. The desalted vegetable material was then drained again using a stainless-steel strainer, pooled, and used to produce laboratory-scale sea fennel pickle prototypes. Salt concentration was measured prior to vinegar addition as described in Section 2.7.4. and reflected the residual NaCl retained in the drained vegetable material after desalination (3.12% NaCl, w w^−1^).

Each prototype consisted of a 500 mL glass jar containing 200 g of desalted sea fennel leaves and stems submerged in 300 mL of pickling solution. Pickling solutions were prepared by diluting commercial apple or wine vinegar (declared acidity 6% and 5%, respectively), purchased from a local market (Ancona, Italy), with mineral water, and supplementing with 3% (w v^−1^) food-grade sucrose, a practice commonly used in industrial pickle production to improve sensory balance by moderating acidity and enhancing overall acceptability of the final product. Dilutions were adjusted to obtain four target acetic acid levels (0.05%, 0.2%, 0.5%, and 0.7%), calculated relative to the total product weight (vegetable material plus pickling solution), in accordance with the minimum safety requirements for acidified vegetable products reported by Breidt [32].

**Figure 3:**
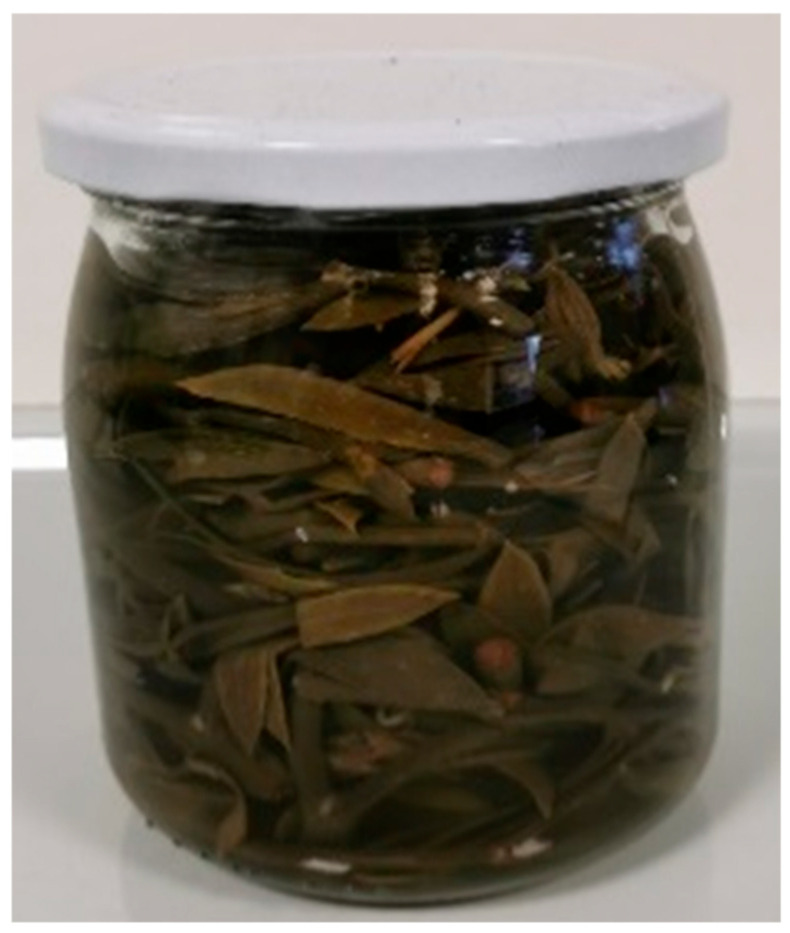
Laboratory-scale prototype of sea fennel pickles.

In batch 1 (B1), three technical replicate jars were prepared for each vinegar–acidity combination, whereas in batch 2 (B2) two technical replicate jars were prepared for each combination (Figure 1). After filling, jars were hermetically sealed and subjected to a mild pasteurization treatment equivalent to 74 °C for 3 min to inactivate vegetative cells and extend shelf life. The jars were then cooled and stored at room temperature (20 ± 2 °C; Figure 3). A six-month monitoring period was conducted for each batch (B1 and B2), during which physicochemical parameters (pH, titratable acidity, salt concentration, and color), microbiological counts, sensory attributes, and volatile profiles were analyzed.

However, based on the results obtained from the microbiological, pH, and titratable acidity analyses, prototypes A1 and W1 (0.05% acidity) in both batches were excluded from subsequent color, salt, sensory, and volatile analyses due to microbial and physico-chemical instability.

### 2.7. Analysis of Laboratory-Scale Prototypes

#### 2.7.1. pH and TA

pH measurements were performed as described in Section 2.4.1. Aliquots (1 mL) of pickling solution were aseptically collected from each jar immediately after pasteurization and at monthly intervals throughout the monitoring period (months 1, 2, 3, 4, 5, and 6). Results were expressed as the mean ± standard deviation of replicates for each batch (B1 and B2).

Titratable acidity (TA) was determined on pickling solution samples aseptically collected immediately after pasteurization (month 0) and at month 6, following the method described in Section 2.4.2. TA values were expressed as the mean ± standard deviation of replicates for each batch (B1 and B2).

#### 2.7.2. Microbial Enumeration

Microbial enumeration was performed on the pickling solution of sea fennel pickles immediately after pasteurization (t_0_) and after 1, 2, 3, 4, 5, and 6 months of storage. Pickling solution samples (1 mL) were aseptically collected from each jar and serially diluted in sterile 0.1% (w v^−1^) peptone water for the enumeration of: (i) mesophilic aerobic bacteria; (ii) presumptive mesophilic lactobacilli; (iii) yeasts and molds; and (iv) Enterobacteriaceae, as described in Section 2.4.3. Viable counts were expressed as the mean ± standard deviation of log colony-forming units (CFU) mL^−1^ of replicates in batches B1 and B2.

In addition, 25 g aliquots consisting of a 1:1 (w w^−1^) mixture of pickled sea fennel leaves and pickling solution were aseptically collected from each jar immediately after pasteurization and at the end of the 6-month monitoring period. These samples were analyzed for: (i) coagulase-positive *Staphylococcus* spp. using the TEMPO: AFNOR BIO 12/28–04/10 standard method; and (ii) sulfite-reducing anaerobic bacteria and spores of sulfite-reducing anaerobic bacteria according to ISO 15213:2003 standard method [33]. The detection of *Listeria monocytogenes* was performed using the miniVIDAS system (bioMérieux, Marcy l’Étoile, France), based on an enzyme-linked fluorescent assay (ELFA), in accordance with AFNOR BIO 12/11–03/04 [34].

#### 2.7.3. Color Assessment

**Figure 4:**
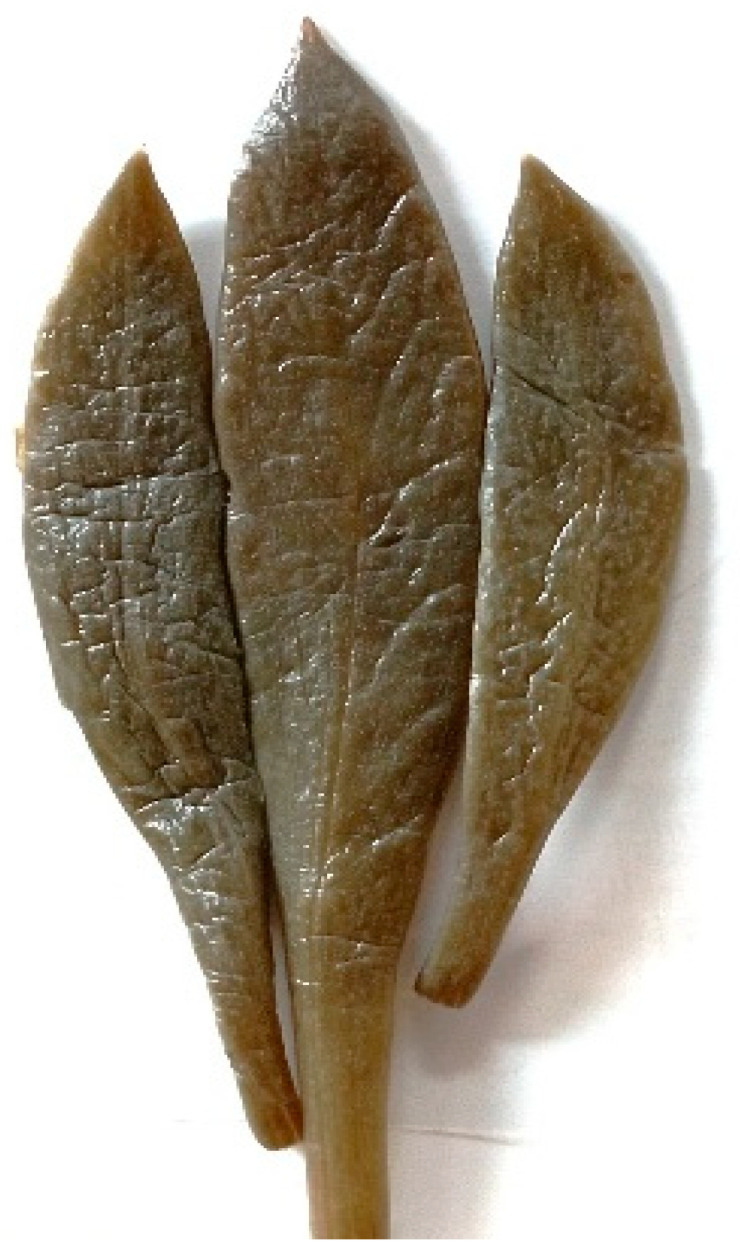
Pickled sea fennel leaves prepared for color analysis.

Color analyss were conducted at the beginning and the end of storage (Months 0, 3 and 6) and performed according to Maoloni et al. [35]. The colorimetric measurements were conducted on sea fennel pickle leaves, grouping together at least three leaves from each jar of similar size to obtain a homogeneous sample (Figure 4).

Color parameters in the CIELAB color space-lightness (L*), redness-greenness (a*: +red; −green), and yellowness-blueness (b*: +yellow; −blue) were measured using a Chroma Meter CR-200 (Minolta, Osaka, Japan). In addition, the hue angle (h°) was calculated using the formula

h° = tan^−1^(b*/a*) + 180°,

Since all samples presented negative a* and positive b* values, corresponding to the second quadrant [36], and the chroma (C*) was calculated as

C = (a*^2^ + b*^2^)^1/2^.

Results were expressed as the mean ± standard deviation of replicates in B1 and B2.

#### 2.7.4. Salt Measurement

NaCl concentration (%, w w^−1^) was determined immediately after desalination of fermented sea fennel leaves and stems and at the beginning and end of storage (months 0 and 6) using a salinity meter (LAQUAtwin Salt-22; HORIBA Ltd., Kyoto, Japan), following the method described by Cardinali et al. [37] with minor modifications. Briefly, sea fennel leaves and stems (5 g) were homogenized with 20 mL of distilled water using an Ultra-Turrax homogenizer (IKA-Werke GmbH & Co., Staufen, Germany) at approximately 10,000 rpm for 2 min to ensure thorough mixing prior to analysis. Results were expressed as the mean ± standard deviation of replicates for each batch (B1 and B2).

#### 2.7.5. Sensory Evaluation

Only microbiologically stable prototypes complying with EU Regulation (EC) No. 2073/2005 (food category “1.3 Ready-to-eat foods unable to support the growth of *Listeria monocytogenes*, other than those intended for infants and for special medical purposes” (Annex I, Chapter 1)) and based on the Italian guidelines on official controls under Regulations (EC) No 882/2004 and 854/2004 (Annex 7, paragraph 22: Preserves, Semi-preserves, and Refrigerated Processed Foods with Extended Shelf Life) were selected for sensory evaluation. Moreover, samples showing fungal growth or microbiological instability were excluded prior to panel evaluation.

Organoleptic properties of laboratory-scale sea fennel pickle prototypes were evaluated on leaves and stems at the end of the monitoring period (t_6_). Sensory evaluation was performed according to the method proposed by Maoloni et al. [7], with minor modifications. Assessments were carried out by a trained panel of eight non-smoking participants (five females and three males, aged 25–52 years). Panelists were preliminarily trained to recognize and describe the sensory attributes of sea fennel pickles through a structured protocol. Briefly, three 60-min training sessions were conducted once a week for three consecutive weeks. In the first week, a variety of sea fennel samples (fresh, blanched, and commercial pickles) were presented to the group to identify and define the key descriptive attributes. During the second week, a consensus discussion was conducted to define the definitive set of descriptors. Regarding the evaluation of the sea fennel pickles, for each prototype, approximately five to six sea fennel leaves were aseptically collected, drained, and placed in blind-coded white plastic cups labeled with random three-digit numbers, then served at room temperature. Evaluations were conducted individually in isolated booths. Still bottled water and plain crackers were provided for palate cleansing between samples.

Samples were evaluated according to the following sensory attributes: (i) olfactory descriptors: herbal odor, woody odor, acidic odor, sea fennel odor; (ii) aroma descriptors: herbal aroma, woody aroma, acidic aroma, sea fennel aroma; (iii) flavor descriptors: acidic flavor, bitterness, salty flavor, sweet flavor; (iv) textural descriptors: hardness, fibrousness, crunchiness; and (v) overall acceptability. The intensity of each descriptor was rated using a 9-point scale (1 = lowest intensity; 9 = highest intensity). Overall acceptability was evaluated using a 9-point hedonic scale, where 1 indicated “extreme dislike” and 9 indicated “extreme like” [38] (P. Results were expressed as the mean ± standard deviation of each descriptor across eight panelists for each prototype in batches B1 and B2.

#### 2.7.6. Volatile Organic Compounds (VOCs) Analysis

Volatile organic compounds (VOCs) were extracted from pickled sea fennel leaves and stems sampled at the beginning (t_0_) and at the end of the storage period (t_6_) in each batch (B1 and B2). For each jar, aliquots of pickled sea fennel leaves and stems (0.5 g) were placed in 10 mL screw-cap vials sealed with PTFE/silicone septa. VOC extraction was performed by headspace solid-phase microextraction (HS-SPME) following the method described by Lenti et al. [39], with minor modifications.

Briefly, vials were equilibrated in a water bath at 40 °C for 10 min, after which headspace volatiles were extracted for 40 min at the same temperature using a divinylbenzene/carboxen/polydimethylsiloxane fiber (DVB/CAR/PDMS, 50/30 µm). The fiber was then thermally desorbed in the injection port of the gas chromatograph at 260 °C for 5 min in splitless mode. Separation and detection were carried out using a Clarus 600 gas chromatograph coupled to a Clarus 600 S mass spectrometer (PerkinElmer, Milan, Italy).

Compounds were separated on a polyethylene glycol-coated capillary column (DB-WAX; J&W Scientific, 60 m × 0.25 mm i.d., 0.25 µm film thickness, Santa Clara, CA, USA). The oven temperature program was as follows: initial temperature of 35 °C (held for 4 min), increased to 120 °C at 2.5 °C min^−1^, then to 250 °C at 15 °C min^−1^, and held for 4 min. Helium was used as the carrier gas at a constant flow rate of 1.2 mL min^−1^. Mass spectra were acquired by electron impact ionization at 70 eV in full-scan mode (m/z 30–400). Data acquisition and processing were performed using TurboMass software (version 5.4.2; PerkinElmer).

VOCs were identified by comparing mass spectra and linear retention indices (calculated using a homologous series of n-alkanes, C_7_–C_30_) with those reported in the NIST Mass Spectral Database, considering only peaks with an intensity threshold of 1.0 × 10^5^. Results were expressed as relative percentages of the total peak area. For statistical analysis, two technical replicates (jars) were considered for each vinegar–acidity combination in batches B1 and B2. The dataset was median-normalized and autoscaled using the MetaboAnalyst 5.0 online platform.

### 2.8. Statistical Analysis

Physicochemical and microbiological data obtained from laboratory-scale sea fennel pickle prototypes during fermentation in brine and after vinegar addition and pasteurization were subjected to one-way analysis of variance (ANOVA). Multiple comparisons among means were performed using the Tukey–Kramer honest significant difference (HSD) test at a significance level of *p* < 0.05, using JMP software (version 11.0.0; SAS Institute Inc., Cary, NC, USA). Partial least squares–discriminant analysis (PLS-DA) was applied to sensory data from prototypes formulated at 0.2% and 0.7% acetic acid in batches B1 and B2. The same prototypes were also analyzed by PLS-DA and heatmap visualization to evaluate differences in volatile profiles between batches, using the MetaboAnalyst 5.0 online platform.

## 3. Results and Discussion

This study evaluated the suitability of sea fennel as a raw material to produce sea fennel pickles with enhanced nutritional and sensory properties, given its high content of bioactive compounds, including polyphenols, carotenoids, vitamins A, C, and E, essential oils, and ω-3 and ω-6 fatty acids [6,40], as well as its proven aptitude for fermentation and preservation [7,28,29]. To this end, sea fennel leaves and stems were processed using a two-step pickling approach. This approach consisted of fermentation in brine followed by vinegar addition at different acidity levels. The following sections report the results obtained during fermentation and those observed after vinegar addition and pasteurization, using two different vinegar types.

### 3.1. Fermentation of Sea Fennel in Brine

This section reports the results of the first pickling step, namely the fermentation of young sea fennel leaves and stems in brine using a multi-strain starter culture. The effects of fermentation were evaluated in terms of physicochemical and microbiological changes occurring during the process, providing the basis for the subsequent vinegar pickling step.

#### 3.1.1. pH Determination

pH is considered an important key quality parameter in fermented preserves, as it significantly influences the sensorial quality, microbial growth, and presents a key indicator of pickle ripeness and potential spoilage [41]. The results of pH of fermented sea fennel in brine are presented in Table 1.

Initially pH values ranged between 6.05 ± 0.02 and 6.28 ± 0.05 in B1 and B2, respectively. During the early stage of fermentation, pH gradually declined in both batches until day 3 with a faster decrease in B2. However, between days 3 and 8, B1 exhibited a faster decrease. Subsequently, from day 8 to 20, pH significantly decreased in both batches reaching significantly lower value in B1 (3.59 ± 0.06) at the end of fermentation.

This acidification pattern is consistent with previous studies on vegetable pickle fermentations inoculated with mixed or selected lactic acid bacteria cultures. Xiong et al. [42] reported a rapid pH decline during pickle fermentation using *Leuconostoc mesenteroides* and *Lactiplantibacillus plantarum*, while McFeeters and Pérez-Díaz [43] observed pH values close to 3.0 after 18 days of cucumber fermentation in NaCl brine inoculated with *L. plantarum*. Comparable trends were also described by Maoloni et al. [29] during the co-fermentation of sea fennel and olives using the same starter culture applied in the present study, with significant pH reductions recorded at the end of fermentation.

The rapid decrease in pH during the early stages of fermentation is particularly relevant for product safety, as it limits the growth of spoilage microorganisms and undesirable microbiota [44]. Acidification further contributes to the inhibition of basophilic bacteria, including coliforms, thereby enhancing microbial stability [45,46]. Similar acidification-driven stabilization has recently been reported by Xia et al. [47] during white radish pickle fermentation, where pH values decreased to 3.91 within 36 h, highlighting the central role of pH reduction in shaping the safety and stability of fermented vegetable products.

#### 3.1.2. TA Determination

Most fermented vegetables undergo acidification as a result of the accumulation of organic acids, mainly lactic and acetic acids, produced by homo- or heterofermentative lactic acid bacteria through the metabolism of free sugars such as glucose and fructose [48].

In the present study, titratable acidity (TA) remained stable in batch B1 throughout fermentation (Table 2), showing no significant increase from day 0 to day 20. In contrast, batch B2 exhibited a significant increase in TA, reaching 0.32 ± 0.03 % lactic acid equivalents at the end of fermentation. A similar behavior was reported by Nilchian et al. [49] during gherkin fermentation, where TA increased during the early stages and stabilized during the late fermentation phase.

During lactic acid fermentation, pH and TA typically exhibit opposite trends, as the accumulation of organic acids results in a progressive decrease in pH [30]. However, batch B1 deviated from this expected pattern, showing a marked pH reduction without a corresponding increase in TA. This behavior may be attributed to the buffering capacity of the plant matrix, which can partially mask changes in titratable acidity despite substantial acidification [50]. In addition, other factors may have contributed to this discrepancy, including variability in the raw plant material and differences in tissue composition. Variations in microbial metabolic activity may also have influenced the acidification dynamics.

Beyond these inherent physiological factors, the distinct chemical profiles of the two seasonal batches served to validate the technological robustness of the process. Specifically, despite a nearly two-fold difference in initial pH values (6.05 vs. 6.28) and TA discrepancy, the starter culture effectively reduced the pH below the target stability threshold of 4.0 in both batches B1 and B2 at the end of fermentation. This outcome underscores the resilience of the acidification protocol and its capacity to ensure product stability regardless of seasonal fluctuations in raw material composition.

Moreover, higher TA levels, as observed in batch B2, are generally associated with enhanced microbial stability through the inhibition of spoilage microorganisms [51].

#### 3.1.3. Viable Counts

**Figure 5:**
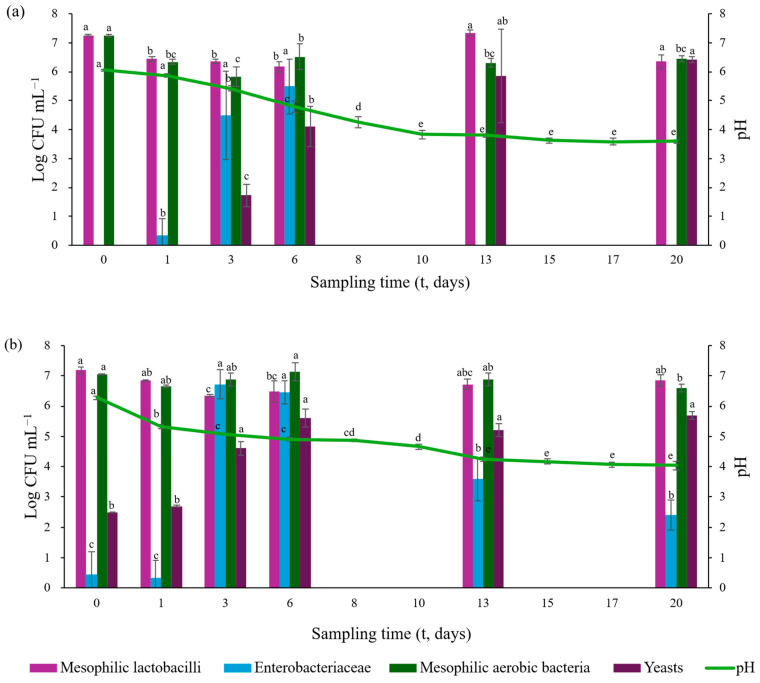
pH and microbiological dynamics of laboratory-scale prototypes of fermented sea fennel in brine. For microbiological analyses, the results are expressed as mean Log CFU mL^−1^ of brine in each batch ± standard deviation, while for pH the results are expressed as means of brine in each batch ± standard deviation (B1, n = 3 biological replicates; B2, n = 3 biological replicates). Panel (**a**) fermented sea fennel in B1; Panel (**b**) fermented sea fennel in B2. For each microbial group and batch, columns labelled with different letters are significantly different (*p* < 0.05). For each sampling time and batch, pH columns labelled with different letters are significantly different (*p* < 0.05).

Viable counts of fermented sea fennel in brine showed batch-dependent variations throughout the fermentation process. Results are reported in Table 3, while the combined trends of pH and microbial populations are illustrated in Figure 5.

The dynamics of lactic acid bacteria observed during fermentation were consistent with the pH trends (Figure 5), reflecting the close relationship between lactic acid bacteria growth, acid production, and pH reduction [52]. In both batches, mesophilic lactobacilli were initially abundant (7.2–7.3 log CFU mL^−1^) and showed a decrease up to day 6, likely due to microbial competition or the antimicrobial activity of sea fennel bioactive compounds [53,54]. Subsequently, lactic acid bacteria populations recovered, reaching by day 13 7.3 ± 0.1 log CFU mL^−1^ and 6.9 ± 0.2 log CFU mL^−1^ in B1 and B2, respectively. This temporary inhibition followed by recovery is consistent with patterns previously described in vegetable fermentations [55] and has also been reported in fermented cowpea pickles [56] and sea fennel brine fermentation using the same starter culture [7]. As expected, lactic acid bacteria played a key role in driving acidification, inhibiting spoilage microorganisms, and contributing to product safety and sensory development [57,58]. The rapid pH decrease observed during the early stages of fermentation likely favored lactic acid bacteria competitiveness, resulting in earlier population stabilization [56].

Mesophilic aerobic bacteria exhibited trends comparable to those of lactic acid bacteria, with high initial counts followed by a reduction during mid-fermentation. In batch B1, a significant decrease reaching 5.8 log CFU mL^−1^ by day 3, followed by stabilization, whereas in batch B2 counts remained relatively stable throughout fermentation (6.6–7.1 log CFU mL^−1^).

Yeasts generally considered undesirable microorganisms in fermented vegetables due to their potential pectinolytic activity and associated textural degradation [59], increased in both batches during fermentation. In B1, yeasts were below the detection limit during the early stages (days 0–3) then increased significantly, reaching 6.7 log CFU mL^−1^ by day 13. In contrast, yeasts were already detectable in B2 at day 0 and exhibited earlier growth, reaching 5.6 log CFU mL^−1^ by day 6. This behavior is consistent with previous reports on sea fennel fermentations [7] and may indicate potential microbial interactions; however, their specific contribution to aroma formation could not be assessed in the absence of VOC analysis during the fermentation stage. The persistence of yeasts at the end of fermentation agrees with findings reported for fermented green olives and sea fennel preserves [7,60,61].

Enterobacteriaceae were below the detection limit (<1.0 log CFU mL^−1^) at t_0_ and t_1_ in both batches. However, detectable levels were observed from day 3 of fermentation, reaching peak values of 4.5 ± 1.5 log CFU mL^−1^ and 6.7 ± 0.5 log CFU mL^−1^ in B1 and B2, respectively. Thereafter, Enterobacteriaceae counts declined markedly by day 20, reaching 2.4 ± 0.5 log CFU mL^−1^ in B2, while they decreased to below detection limit in B1. This reduction is attributable to the acidic conditions generated by lactic acid bacteria activity and reflects the typical microbial succession observed in vegetable fermentations [7,52,62]. In addition, the blanching step applied prior to fermentation was selected as a treatment commonly applied to fresh vegetables to reduce microbial load [7]. This step may have contributed to the early reduction of Enterobacteriaceae in accordance with previous findings [7,29], as this treatment is recognized as a processing operation capable of reducing microbial spoilage, including microorganisms potentially responsible for food spoilage or foodborne illness [63,64], although without significantly affecting yeast survival. Spore-forming bacteria and molds were not detected in either batch throughout fermentation.

Overall, despite batch-specific differences, fermentation of sea fennel in brine followed a typical microbial succession characterized by lactic acid bacteria dominance, transient growth of Enterobacteriaceae followed by inhibition, and yeast enrichment accompanying progressive pH reduction (Figure 5).

### 3.2. Laboratory-Scale Prototypes of Sea Fennel Pickle Preserves

Fermentation of sea fennel in brine represented a key step in pickle manufacturing, as it promoted the growth of lactic acid bacteria, reduced pH, and enriched the product with desirable metabolites, thereby improving both safety and nutritional quality. This biologically driven acidification resulted in a stable fermented matrix suitable for subsequent processing [7]. Building on these outcomes, fermented sea fennel was further processed into vinegar-treated pickles using two different vinegar types (wine and apple) at four acetic acid levels (0.05%, 0.2%, 0.5%, and 0.7%), resulting in four distinct prototypes for each vinegar type.

In addition to fermentation, vinegar provides acetic acid, which enables rapid acidification, imparts distinctive aromatic properties, and exerts a strong inhibitory effect against pathogenic microorganisms such as *Escherichia coli* and *Clostridium botulinum* [65]. To further enhance product stability and safety, a mild pasteurization treatment was applied to ensure extended shelf life through the inactivation of residual microbial populations [66], while preserving textural and sensory attributes of the final product [67].

#### 3.2.1. Determination of pH and TA

The pH and titratable acidity (TA) values of pickled sea fennel prototypes are reported in Table 4 and Table 5, respectively. Overall, pH values showed limited variation throughout the six-month storage period, except for samples A1 and W1. Slightly greater pH stability was observed in batch B2 compared with batch B1. Differences between batches were unlikely to compromise product stability. As expected, higher acidity levels were consistently associated with lower pH values, regardless of vinegar type. Across all formulations, pH values ranged from 3.39 to 3.83 in B1 and from 3.51 to 4.37 in B2, in agreement with previously reported values for pickled sea fennel products [28].

Compared with pH values measured after fermentation (approximately 3.6), higher pH values were generally observed in low-acidity formulations (W1 and A1) following pasteurization. This increase may be attributed to the combined effects of washing, prolonged storage, and thermal treatment. Moreover, during storage, samples prepared with the lowest acidity level (0.05%) in batch B1 exhibited a progressive decrease in pH, whereas only minor changes were observed in the corresponding samples from batch B2. Such differences may reflect batch-dependent variability in raw materials or processing conditions, as well as the possible activity of spoilage microorganisms capable of tolerating acidic environments.

Similarly, during storage increases in TA were observed in some low-acidity prototypes (W1 and A1), consistent with the concomitant pH decrease, whereas other formulations remained stable. In both batches, prototypes prepared with an intermediate acidity level (0.2%; W2 and A2) showed the greatest stability in terms of both pH and TA throughout storage, in agreement with previous observations reported for pickled vegetable products [68].

The final pH and titratable acidity values reflect the combined effect of fermentation and subsequent technological steps, including vinegar addition at different concentrations and pasteurization for product stabilization. As a result, final acidity is largely influenced by the controlled acidification applied during processing, which may partially reduce differences arising from the fermentation phase. Moreover, acidity stability was influenced by formulation and batch characteristics, with intermediate acidity levels (0.2%) providing the most consistent behavior. This finding is particularly relevant for product standardization and shelf-life control in the development of fermented sea fennel pickles.

#### 3.2.2. Microbiological Analysis

Understanding and monitoring microorganisms that may compromise food quality and safety remains a priority for the food sector and regulatory authorities [69]. The microbiological results obtained for sea fennel pickle prototypes during six-months storage are reported in Table 6.

Overall, most microbial groups remained below the detection limit (<1 log CFU mL^−1^) throughout the storage period, except yeasts and mesophilic aerobic bacteria. Mesophilic lactobacilli were not detected in either batch, except for prototype A2 in batch B2 from t_1_, where low counts were recorded before declining to undetectable levels by t_6_. As this occurrence was observed in only one replicate, it is likely attributable to handling or sampling variability. These findings confirm the effectiveness of pasteurization in inactivating lactic acid bacteria and enhancing the microbiological safety of acidified products such as pickles.

Enterobacteriaceae were not detected in any prototype at any sampling time (<1 log CFU mL^−1^), confirming the microbiological safety of the products during storage. As reported in Section 3.1.3, Enterobacteriaceae were present at the end of fermentation but were completely inhibited after pasteurization, with no regrowth observed during storage. Given that this microbial group is commonly used as an indicator of hygiene rather than product quality [70], these results further support the effectiveness of pasteurization even under controlled acidic conditions, as previously reported for pickled vegetables [71].

Mesophilic aerobic bacteria remained undetectable in most prototypes; however, samples prepared with the lowest acidity levels exhibited progressive growth during storage. In batch B2, microbial growth was detected mainly in A1 and A2, while W1 remained mostly below the detection limit throughout storage. As mesophilic aerobic bacteria are commonly used as indicators of food quality and spoilage [70], their increase in low-acidity formulations may have contributed to the changes in pH and titratable acidity observed during storage [72,73].

Yeast growth was particularly evident in low-acidity prototypes (A1 and W1). In batch B1, yeast counts reached approximately 3.5 log CFU mL^−1^ by t_6_, whereas in batch B2 exhibited lower levels, with partial inhibition in prototype A2 by the end of storage. These findings are consistent with previous observations reported for pickled vegetables [12]. Although yeasts were initially reduced by pasteurization, their regrowth during storage may be favored by low acidity and suitable environmental conditions, potentially contributing to texture degradation, as described in earlier studies [74,75].

Molds generally remained below the detection limit, except for prototype W1 in batch B1, which showed microbial growth at t_2_ and t_3_. In addition, visible surface growth consistent with filamentous fungi was observed in some low-acidity samples (A1 and W1) in both batches during storage. Owing to the high dispersion of mold counts in W1 of B1 and the nature of the dataset (n = 3), statistical groupings are reported to support data description. This apparent discrepancy may be explained by the heterogeneous and surface-localized nature of mold growth in pickled products, which may not be adequately captured by brine sampling alone [76]. In this context, the concurrent growth of yeasts and aerobic mesophilic bacteria in low-acidity prototypes further indicates reduced microbial stability and conditions conducive to surface spoilage.

Sulfite-reducing anaerobes (including spores) were not detected in any prototype at the beginning or at the end of the storage period. Coagulase-positive *Staphylococcus* spp. and *Listeria monocytogenes* were also not detected in any prototype at the end of storage. Overall, pickle quality was considered safe and acceptable according to ICMSF criteria [77].

Taken together, these results confirm that fermentation followed by vinegar addition and pasteurization effectively controls microbial populations in sea fennel pickles. However, prototypes prepared with the lowest acidity levels (A1 and W1) in both batches exhibited microbial instability and visible product deterioration during storage and were therefore excluded from subsequent analyses.

#### 3.2.3. Color Analysis

Color, a key determinant of consumer acceptance of pickled products [15], was monitored in sea fennel pickles during storage using the CIELAB color system. Color parameters (L*, a*, b*, chroma, C, and hue angle, h°) are reported in Table 7.

In batch B1, lightness (L*) remained generally stable throughout storage, except for an increase observed in prototype A2 at t_6_ and a slight decrease in W4. While this shift in A2 indicates a possible lightning of the samples, the simultaneous stability of the Hue angle h° and Chroma C suggests that the core pigment identity was preserved.

In contrast, batch B2 exhibited more consistent L* values over time. Minimal variation was observed along the a* coordinate; however, significant decreases were detected in prototypes A4 (B1) and W4 and A2 (B2). Samples from batch B2 generally retained greener tones compared with those from batch B1.

Regarding the b* coordinate, most prototypes in batch B1 showed a significant decrease at the end of the storage period, whereas batch B2 maintained higher b* values. Hue angle values remained within the yellow–green quadrant (h° > 100°) throughout storage in all formulations. The chroma index decreased in most prototypes, particularly those prepared with wine vinegar, although batch B2 retained higher color saturation over time. In contrast to the findings reported by Radman et al. [28], higher green intensity was not observed in samples prepared with wine vinegar in the present study.

Color losses observed in high-acidity prototypes (0.7%) may be associated with acid-induced oxidation of phytochemicals, as previously suggested [78]. Processing and storage-related effects on pigment stability have been widely reported [79,80], and the decreases in L* and a* observed here are consistent with those reported for acidified vegetables by Zhao et al. [81].

Overall, batch B2 showed greater color stability compared with batch B1. These differences may be related to variability in raw materials or processing conditions. Apple vinegar prototypes, particularly A2, showed greater color stability than those prepared with wine vinegar, highlighting the influence of vinegar type and acidity level on the visual quality of sea fennel pickles.

#### 3.2.4. Salt Determination

Salt plays a key role in pickle production by regulating fermentation and contributing to flavor development through the formation of acids, aldehydes, alcohols, and esters [15,82]. In addition, salt generates osmotic pressure that promotes the release of intracellular sugars, thereby supporting lactic acid bacteria activity during fermentation [83]. Salt concentration results are reported in Table 8.

At t_0_, no significant differences were observed between batches. By the end of the storage period, salt content significantly decreased in most samples in both batches, except for prototype W2 in B2. Final salt concentrations ranged from 1.05 ± 0.00 to 1.70 ± 0.28% NaCl in B1 with comparable values were observed in B2, although W2 retained a higher concentration (2.00% NaCl). Overall, neither vinegar type nor batch significantly affected final salt concentrations.

These results are consistent with salt levels previously reported for vinegar pickles and fermented vegetables [28,83]. Salt concentrations around 1% NaCl have been shown to improve the chemical and sensory properties of fermented vegetables when combined with lactic acid fermentation [84]. In line with World Health Organization recommendations to reduce dietary salt intake [85], fermentation represents an effective strategy to lower salt levels without compromising product safety or quality [86]. This approach has also been demonstrated to be effective under low-salt conditions using *Lactiplantibacillus plantarum* as a starter culture [59].

#### 3.2.5. Sensory Analysis

**Figure 6:**
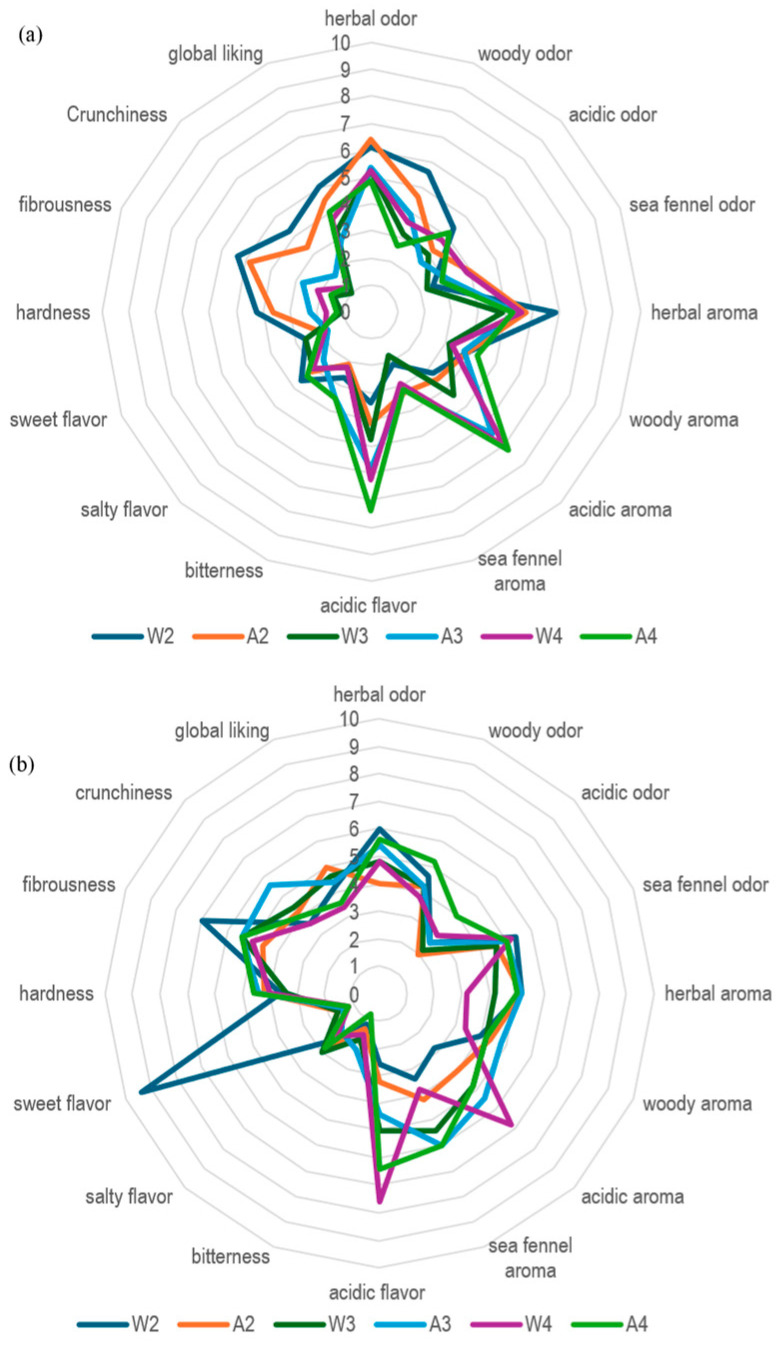
Sensory analysis of leaves and stems of laboratory-scale sea fennel pickle prototypes in B1 and B2. (**a**) Sensory analyses performed in B1; (**b**) Sensory analyses performed in B2. Each sample was evaluated by a trained panel, consisting of 8 non-smoker tasters aged between 25 and 48, (i) olfactory descriptors: herbal odor, woody odor, acidic odor, sea fennel odor; (ii) aroma descriptors: herbal aroma, woody aroma, acidic aroma, sea fennel aroma; (iii) flavor descriptors: acidic flavor, bitterness, salty flavor, sweet flavor; (iv) textural descriptors: hardness, fibrousness, crunchiness; (v) overall acceptability. Each descriptor was evaluated by attributing a score comprised between 1 and 9, with 1 expressing the lowest and 9 the highest intensity. Results are reported as mean of pickles sea fennel leaves and stems ± standard deviation. For legend prototypes see Table 4.

Pickles are commonly evaluated based on sensory attributes such as taste, texture, aroma, flavor, and aftertaste [87]. Previous studies have shown that fermented vegetable pickles generally achieve higher consumer acceptability than fresh vegetables [88]. Accordingly, sensory analysis was performed at the end of the storage period (t_6_) to evaluate the effects of acidity level and storage on sea fennel pickle prototypes (Figure 6). Prototypes W1 and A1 were excluded from sensory evaluation due to texture degradation associated with microbial growth as previously reported in Section 2.7.5 and Section 3.2.2. Only prototypes that did not show microbial instability and met the safety requirements of Regulation (EC) No. 2073/2005 and Regg. CE 882/04 e 854/04 of Italian guidelines at the end of storage were subjected to sensory analysis so prototypes A2, A3, A4 and W2, W3, W4 in both batches B1 and B2.

Overall, most sea fennel pickle prototypes received high scores for acidic flavor and aroma, reflecting the combined contribution of vinegar addition and intrinsic sea fennel phytochemicals. Some variability was observed between batches, with batch B2 exhibiting greater texture stability, possibly related to differences in raw material quality, processing conditions, or storage parameters. In batch B1, prototypes W2 and A2 showed higher hardness and fibrousness scores, indicating better preservation of textural attributes.

Sea fennel odor and aroma were more pronounced in prototypes formulated with higher acidity levels (0.5% and 0.7%), particularly in batch B2, suggesting improved preservation of characteristic sensory traits. However, increasing acidity intensified sourness and negatively affected overall acceptability, likely due to excessive acidic flavor and increased leaf hardness, while simultaneously reducing herbal and woody notes. In addition to acidity-related effects, pasteurization may have further influenced sensory quality, particularly color, flavor, and texture, as previously reported for acidified vegetable products [89,90]. Moreover, the addition of 3% sucrose may have contributed to the balanced sensory profile of the prototypes by moderating the acidity of the vinegar. This sugar-to-acid balance is a common technological approach in the production of mild vegetable preserves to enhance the flavor and texture during storage.

Wine vinegar at 0.2% acidity was preferred in batch B1 and apple vinegar at the same acidity level being favored in batch B2. Nevertheless, vinegar type had a limited influence on individual sensory attributes. Taken together, these results indicate that a moderate acidity level (0.2%) provides a favorable balance between sensory quality and overall product acceptability for fermented sea fennel pickles.

#### 3.2.6. Multivariate Analysis

**Figure 7:**
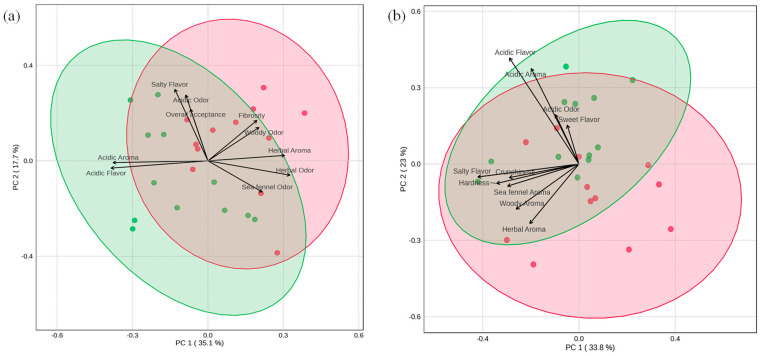
Partial least squares-discriminant analysis (PLS-DA) of sensory attributes of laboratory-scale sea fennel pickle prototypes in batch 1 and batch 2. Panel (**a**): Batch 1; Panel (**b**): Batch 2. Red ellipse and dots = 0.2% acidity prototypes; green ellipse and dots = 0.7% acidity prototypes.

Based on preliminary results, prototypes formulated with 0.2% acetic acid exhibited the most favorable balance between microbial stability and sensory quality. Accordingly, partial least squares–discriminant analysis (PLS-DA) was applied (Figure 7) to samples prepared at 0.2% and 0.7% acidity in batches B1 and B2 to visualize sensory differentiation between the two acidity levels, independently of vinegar type. Prototypes at 0.7% acidity were included to explore sensory shifts associated with higher acidity, despite their lower overall acceptability.

In batch B1 (Figure 7a), samples preserved at 0.2% (red) and 0.7% (green) acidity showed partial overlap but were characterized by distinct sensory drivers. Fibrousness, woody odor, and herbal aroma were more strongly associated with the 0.2% acidity level, whereas acidic flavor and acidic aroma were positively correlated with the higher acidity. Overall liking was positioned closer to the 0.2% prototypes, indicating that these attributes contributed positively to consumer preference.

Similarly, prototypes from batch B2 (Figure 7b) were clearly distinguished based on their vinegar concentration. Specifically, samples formulated at 0.7% acidity were associated with acidic and sweet flavor notes, whereas those at 0.2% acidity correlated with hardness, crunchiness, sea fennel odor, and herbal and woody aromas. Taken together, these results confirm that vinegar concentration is a primary factor driving sensory differentiation in sea fennel pickle prototypes, with moderate acidity levels favoring attributes associated with higher overall acceptability.

#### 3.2.7. Volatile Organic Compounds (VOCs) Determination

**Figure 8:**
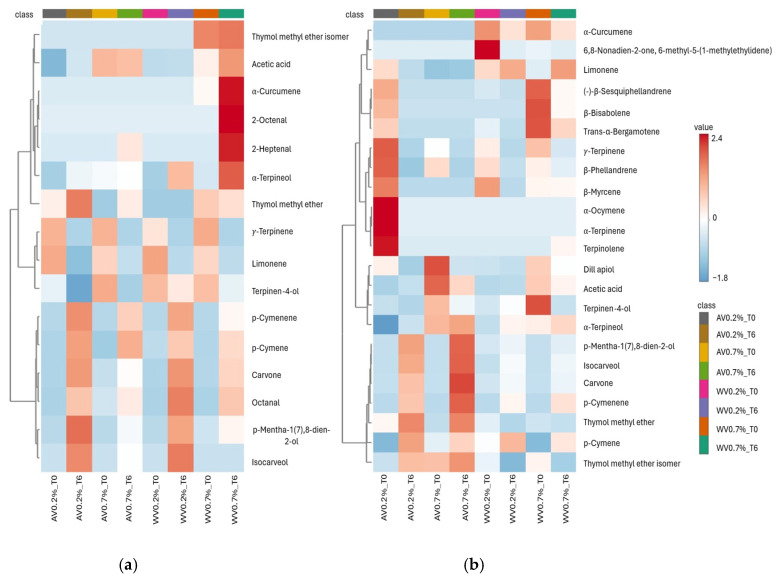
Hierarchical clustering heatmap of volatile organic compounds in leaves and stems of laboratory-scale sea fennel pickle prototypes, preserved with apple or wine vinegar at 0.2% and 0.7% acidity. Panel (**a**): batch 1; Panel (**b**): batch 2. AV0.2 = apple vinegar prototype with 0.2% acidity; AV0.7 = apple vinegar prototype with 0.7% acidity; WV0.2 = wine vinegar prototype with 0.2%; WV0.7% = wine vinegar prototype with 0.7% acidity, sampling time: T0 = Month 0; T6= Month 6. Cell colours indicate the relative percentage of the total volatile content for each VOC, blue representing low concentration and red high concentration.

**Figure 9:**
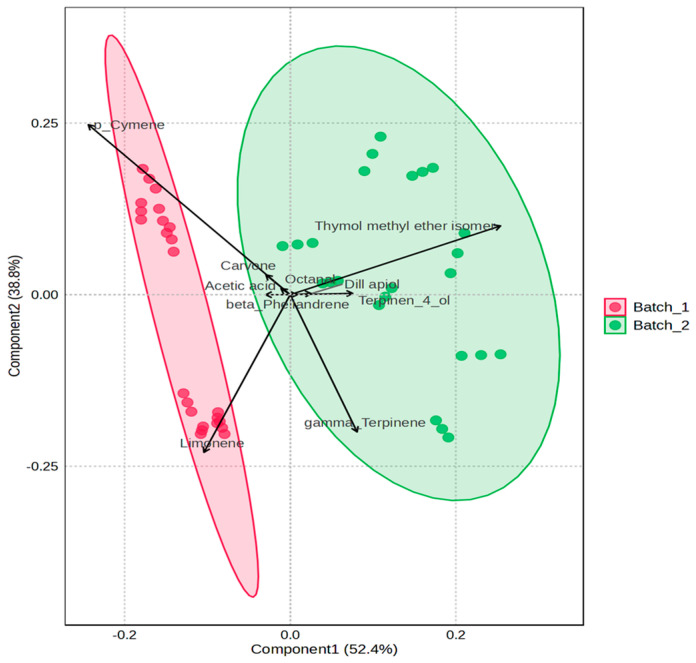
Partial least squares-discriminant analysis (PLS-DA) of VOC profile in leaves and stems of the laboratory-scale sea fennel pickle prototypes from batch 1 and batch 2. Red ellipse and dots = Batch 1; green ellipse and dots = Batch 2.

VOC analysis was performed on the same group of prototypes formulated at 0.2% and 0.7% acidity (W2, W4, A2, and A4) in both batches B1 and B2, as described in Section 3.2.6, to investigate the effects of acidity level and storage on volatile profiles. The volatile organic compound composition of sea fennel pickle leaves and stems preserved with apple or wine vinegar at 0.2% and 0.7% acidity in batches B1 and B2 is reported in Table 9 and Table 10, respectively. Figure 8 illustrates the relative distribution of the main volatile compounds at the beginning (t_0_) and end (t_6_) of storage using a heatmap, while Figure 9 presents the partial least squares–discriminant analysis (PLS-DA) comparing the two batches.

Overall, 16 VOCs were identified in batch B1 and 23 VOCs in batch B2. Despite these quantitative differences, both batches shared a core volatile profile dominated by monoterpenes, particularly limonene (16.51–50.09%), γ-terpinene (10.71–28.91%), and p-cymene (12.80–34.62%), which are characteristic of sea fennel aroma. However, the evolution of these compounds during storage differed markedly between batches (Figure 8).

In batch B1, limonene and γ-terpinene showed pronounced degradation during storage, particularly in prototypes formulated at 0.7% acidity. In apple vinegar samples, γ-terpinene decreased to undetectable levels by t_6_. In contrast, p-cymene increased over time in both apple and wine vinegar samples, while limonene generally declined (Figure 8a). At t_6_, wine vinegar prototypes at both acidity levels exhibited an increase in several compounds, including α-curcumene, 2-octenal, 2-heptenal, and α-terpineol. Although present at low relative abundances, these compounds were detected exclusively at the end of storage (Table 9), suggesting storage- and acidity-driven transformations of terpene precursors.

By comparison, batch B2 exhibited greater terpene stability throughout storage (Figure 8b). γ-Terpinene was partially retained, reaching 5.99% at t_6_ in wine vinegar samples at 0.7% acidity. Moreover, limonene increased in wine vinegar prototypes at both acidity levels, rising from 27.67% to 31.81% at 0.2% acidity and from 21.93% to 32.96% at 0.7% acidity. In both apple and wine vinegar samples formulated at 0.7% acidity, terpinen-4-ol was abundant at t_0_ (10.27% and 14.41%, respectively) then decreased by the end of storage to 6.30% and 4.66%, respectively. In addition, the thymol methyl ether isomer increased over time in apple vinegar prototypes at 0.2% acidity, rising from 19.47% to 27.46% (Table 10). These observations are consistent with those reported by Radman et al. [28], who identified terpinen-4-ol among the major volatile components of vinegar-preserved sea fennel.

Further batch-related differences were observed for sesquiterpenes and phenolic derivatives. In batch B2, sesquiterpenes such as α-curcumene were better preserved in wine vinegar samples than in apple vinegar samples, whereas these compounds were negligible in batch B1. Dill apiol was detected exclusively in batch B2 and decreased during storage, while carvone appeared in trace amounts at t_6_, indicating ongoing compositional changes during storage.

These trends are supported by previous studies on sea fennel volatile compounds. Özcan et al. [91] reported sabinene, limonene, and γ-terpinene as dominant volatiles in fresh sea fennel, with decreases during fermentation accompanied by increases in terpinen-4-ol and related oxygenated terpenes. Moreover, chemotype variability has been widely documented: Raffo et al. [92] demonstrated that sea fennel essential oil composition varies according to multiple factors influencing sea fennel composition, including genetic background, environmental conditions, plant organ analyzed, phenological stage, harvest period, and geographical origin, while Politeo et al. [93] reported marked differences between French and Croatian ecotypes, characterized by dill apiol–rich and limonene/sabinene-rich profiles, respectively. These factors contribute to the natural chemotypes diversity observed in sea fennel. Therefore, the volatile composition of sea fennel pickle preserves appears to be influenced not only by acidity level, storage conditions, and vinegar type, but reflects intrinsic raw material variability together with processing-related factors.

Moreover, the observed divergence between the volatile profile of the vinegar prototypes and their limited sensory differentiation highlights the complexity of flavor perception. Aroma perception is not a direct reflection of volatile abundance but depends on factors such as the dynamic release of compounds from the food matrix and perceptual interactions among volatiles, including masking and synergistic effects [94,95]. In this context, it is plausible that the dominant monoterpenes characteristic of sea fennel (e.g., limonene) may have partially masked more subtle aromatic differences associated with vinegar type, resulting in a relatively consistent sensory profile despite measurable variations in volatile composition. However, this interpretation remains hypothetical in the absence of Odor Activity Value (OAV) calculations.

PLS-DA confirmed that the two batches exhibited distinct volatile profiles, with specific compounds driving the observed discrimination. The minimal overlap between the confidence ellipses highlights the high reproducibility within each batch (Figure 9).

A clear separation between batches B1 and B2 was observed based on the volatile profiles of pickled sea fennel.

Component 1, accounting for 52.4% of the total variance, captured the main differences in volatile composition between the two batches, while Component 2 explained an additional 38.8% of the variance, further contributing to sample discrimination. The shaded ellipses represent the 95% confidence regions and indicate limited intra-batch variability.

Inspection of the loading vectors revealed the volatile compounds primarily responsible for batch separation. Batch B1 was associated with higher relative abundances of p-cymene, limonene, and carvone, whereas batch B2 was characterized by elevated levels of thymol methyl ether isomer, dill apiol, γ-terpinene, and terpinen-4-ol. In contrast, compounds such as acetic acid, octanal, and β-phellandrene were positioned close to the origin, suggesting a limited contribution to batch discrimination.

## 4. Conclusions

The findings of this study provide a comprehensive evaluation of a combined preservation strategy based on fermentation, vinegar addition, and pasteurization to produce high-quality sea fennel preserves with enhanced storage stability. During fermentation, pH showed a progressive decrease to values below 3.6, confirming effective biological acidification that ensured microbial stability and was not significantly influenced by batch variability. Lactic acid bacteria dominated the final stages of fermentation following initial fluctuations, leading to a favorable microbial succession characterized by the inhibition of Enterobacteriaceae and enrichment of their populations. Yeasts showed increasing growth, while spore-forming bacteria were not detected.

The evaluation of pickle prototypes during storage indicated that pH, titratable acidity, and salt concentration were generally stable in most prototypes and were not significantly affected by vinegar type or batch. Salt concentrations slightly decreased during storage but remained within a safe and acceptable range. However, microbiological analyses revealed growth of yeasts and mesophilic aerobic bacteria in prototypes prepared with the lowest acidity level (0.05%), demonstrating that this acidity level does not ensure adequate product stability, regardless of vinegar type or pasteurization.

Sensory analysis demonstrated that a moderate acidity level (0.2%) provided the best compromise between texture preservation, characteristic sea fennel aroma, and overall acceptability. Volatile compound analysis confirmed the dominance of monoterpenes such as limonene, γ-terpinene, and *p*-cymene, with batch B2 and wine vinegar prototypes showing better preservation of key aroma compounds during storage.

Overall, this study demonstrates that the combination of controlled lactic fermentation, vinegar pickling at moderate acidity of 0.2%, and pasteurization can produce microbiologically stable, sensorially acceptable, and shelf-stable sea fennel pickles under the conditions tested. This integrated approach represents a promising basis for the development of vegetable preserves and may support future scale-up and industrial applications. The results also support the interest in sea fennel as a halophytic crop for further exploration in the context of sustainable utilization of plants adapted to saline environments.

However, certain limitations must be acknowledged. The PLS-DA analysis of VOC profiles (B1 and B2) showed that samples clustered primarily according to batch, confirming that raw material variability, likely associated with seasonal chemotypes, remains the primary driver of the final aromatic traits. While the proposed protocol ensures microbial safety and practical stability, it does not fully eliminate the influence of raw material variability on sensory characteristics. Therefore, further studies including a wider range of harvests, cultivation conditions, and larger-scale production trials are needed to improve process standardization and ensure consistent quality of sea fennel pickles.

## Figures and Tables

**Table 1:** Results of pH measurements in laboratory-scale prototypes of fermented sea fennel in brine.

Sampling Time (t, Days)	Fermented Sea Fennel in Brine	
	B1	B2
t_0_	6.05 ± 0.02 ^a,B^	6.28 ± 0.05 ^a,A^
t_1_	5.86 ± 0.06 ^a,A^	5.32 ± 0.07 ^b,B^
t_3_	5.43 ± 0.10 ^b,A^	5.08 ± 0.03 ^c,B^
t_6_	4.78 ± 0.19 ^c,A^	4.91 ± 0.05 ^c,A^
t_8_	4.25 ± 0.20 ^d,B^	4.86 ± 0.04 ^cd,A^
t_10_	3.83 ± 0.16 ^e,B^	4.66 ± 0.09 ^d,A^
t_13_	3.80 ± 0.05 ^e,B^	4.25 ± 0.08 ^e,A^
t_15_	3.62 ± 0.08 ^e,B^	4.17 ± 0.09 ^e,A^
t_17_	3.58 ± 0.12 ^e,B^	4.07 ± 0.09 ^e,A^
t_20_	3.59 ± 0.06 ^e,B^	4.04 ± 0.14 ^e,A^

Values are expressed as means ± standard deviations of brine samples in each batch (B1, n = 3 biological replicates; B2, n = 3 biological replicates). Superscripts indicate statistical significance according to ANOVA and Tukey’s post hoc test. Superscript lowercase letters indicate statistically significant differences between samples within the same column (*p* < 0.05), while superscript uppercase letters indicate statistically significant differences between samples within the same row (*p* < 0.05).

**Table 2:** Results of TA determination of laboratory-scale prototypes of fermented sea fennel in brine.

Sampling Time (t, Days)	Fermented Sea Fennel in Brine	
	B1	B2
t_0_	0.12 ± 0.04 ^a,A^	0.03 ± 0.00 ^b,B^
t_20_	0.18 ± 0.02 ^a,B^	0.32 ± 0.03 ^a,A^

Values are expressed as mean ± standard deviation of % lactic acid equivalents in brine samples (B1, n = 3 biological replicates; B2, n = 3 biological replicates). Superscripts indicate statistical significance according to ANOVA and Tukey’s post hoc test. Superscript lowercase letters indicate statistically significant differences between samples within the same column (*p* < 0.05), while superscript uppercase letters indicate statistically significant differences between samples within the same row (*p* < 0.05).

**Table 3:** Results of microbiological counting of laboratory-scale prototypes of fermented sea fennel in brine.

Microbial Group	Sampling Time (t, Days)	Batch	
		B1	B2
Mesophilic lactobacilli (Log CFU mL^−1^)			
	t_0_	7.3 ± 0.0 ^a,A^	7.2 ± 0.1 ^a,A^
	t_1_	6.4 ± 0.1 ^b,B^	6.8 ± 0.0 ^ab,A^
	t_3_	6.4 ± 0.1 ^b,A^	6.3 ± 0.0 ^c,A^
	t_6_	6.2 ± 0.2 ^b,A^	6.5 ± 0.4 ^bc,A^
	t_13_	7.3 ± 0.1 ^a,A^	6.7 ± 0.2 ^abc,B^
	t_20_	6.4 ± 0.2 ^a,A^	6.9 ± 0.2 ^ab,B^
Yeasts (Log CFU mL^−1^)			
	t_0_	<1.0 ^c,B^	2.5 ± 0.2 ^b,A^
	t_1_	<1.0 ^c,B^	2.7 ± 0.1 ^b,A^
	t_3_	1.7 ± 0.4 ^c,B^	4.6 ± 0.4 ^a,A^
	t_6_	4.1 ± 0.7 ^b,B^	5.6 ± 0.1 ^a,A^
	t_13_	5.9 ± 1.6 ^ab,A^	5.2 ± 1.2 ^a,A^
	t_20_	6.4 ± 0.1 ^a,A^	5.7 ± 0.2 ^a,B^
Molds (Log CFU mL^−1^)			
	t_0_	<1.0	<1.0
	t_1_	<1.0	<1.0
	t_3_	<1.0	<1.0
	t_6_	<1.0	<1.0
	t_13_	<1.0	<1.0
	t_20_	<1.0	<1.0
Enterobacteriaceae (Log CFU mL^−1^)			
	t_0_	<1.0 ^b,A^	<1.0 ^c,A^
	t_1_	<1.0 ^b,A^	<1.0 ^c,A^
	t_3_	4.5 ± 1.5 ^a,A^	6.7 ± 0.5 ^a,A^
	t_6_	5.5 ± 0.9 ^a,A^	6.5 ± 0.4 ^a,A^
	t_13_	<1.0 ^b,B^	3.6 ± 0.7 ^b,A^
	t_20_	<1.0 ^b,B^	2.4 ± 0.5 ^b,A^
Mesophilic aerobic bacteria (Log CFU mL^−1^)			
	t_0_	7.3 ± 0.0 ^a,A^	7.1 ± 0.0 ^a,B^
	t_1_	6.3 ± 0.1 ^bc,B^	6.7 ± 0.0 ^ab,A^
	t_3_	5.8 ± 0.3 ^c,B^	6.9 ± 0.2 ^ab,A^
	t_6_	6.5 ± 0.4 ^b,A^	7.1 ± 0.3 ^a,A^
	t_13_	6.3 ± 0.2 ^bc,B^	6.9 ± 0.2 ^ab,A^
	t_20_	6.5 ± 0.1 ^bc,A^	6.6 ± 0.1 ^b,A^
Spore-forming bacteria (Log CFU mL^−1^)			
	t_0_	<1.0	<1.0
	t_1_	<1.0	<1.0
	t_3_	<1.0	<1.0
	t_6_	<1.0	<1.0
	t_13_	<1.0	<1.0
	t_20_	<1.0	<1.0

Values are expressed as mean Log CFU mL^−1^ of brine ± standard deviations in each batch (B1, n = 3 biological replicates; B2, n = 3 biological replicates). Superscripts indicate statistical significance according to ANOVA and Tukey’s post hoc test. Superscript lowercase letters indicate statistically significant differences between samples within the same column (*p* < 0.05), while superscript uppercase letters indicate statistically significant differences between samples within the same row (*p* < 0.05).

**Table 4:** Results of pH measurements in laboratory-scale sea fennel pickle prototypes.

Batch	Sampling Time (t. Months)	Prototypes							
		W1	W2	W3	W4	A1	A2	A3	A4
B1	t_0_	4.32 ± 0.02 ^a,B^	3.75 ± 0.01 ^a,B^	3.39 ± 0.01 ^c,B^	3.28 ± 0.01 ^c,B^	4.36 ± 0.02 ^a,B^	3.79 ± 0.02 ^c,B^	3.52 ± 0.14 ^c,B^	3.40 ± 0.01 ^a,B^
	t_1_	4.00 ± 0.08 ^ab,B^	3.53 ± 0.04 ^b,B^	3.17 ± 0.05 ^d,B^	3.11 ± 0.01 ^d,B^	3.98 ± 0.02 ^b,B^	3.57 ± 0.02 ^c,B^	3.27 ± 0.12 ^d,B^	3.23 ± 0.15 ^b,B^
	t_2_	3.95 ± 0.10 ^ab,B^	3.75 ± 0.01 ^a,B^	3.43 ± 0.02 ^bc,B^	3.34 ± 0.04 ^bc,B^	4.11 ± 0.08 ^ab,A^	3.71 ± 0.02 ^d,A^	3.48 ± 0.07 ^c,B^	3.41 ± 0.01 ^a,A^
	t_3_	3.92 ± 0.14 ^ab,B^	3.79 ± 0.03 ^a,B^	3.50 ± 0.08 ^abc,A^	3.40 ± 0.01 ^ab,B^	4.10 ± 0.12 ^ab,A^	3.81 ± 0.03 ^bc,B^	3.61 ± 0.02 ^ab,A^	3.45 ± 0.01 ^a,B^
	t_4_	3.87 ± 0.22 ^ab,A^	3.80 ± 0.04 ^a,A^	3.59 ± 0.02 ^a,A^	3.42 ± 0.03 ^a,B^	4.09 ± 0.25 ^ab,A^	3.90 ± 0.03 ^a,A^	3.64 ± 0.03 ^a,A^	3.49 ± 0.01 ^a,A^
	t_5_	3.82 ± 0.20 ^ab,A^	3.79 ± 0.03 ^a,B^	3.53 ± 0.06 ^ab,B^	3.39 ± 0.01 ^ab,B^	3.75 ± 0.21 ^bc,B^	3.87 ± 0.02 ^ab,B^	3.60 ± 0.02 ^ab,B^	3.48 ± 0.02 ^a,B^
	t_6_	3.73 ± 0.34 ^b,A^	3.77 ± 0.01 ^a,B^	3.47 ± 0.03 ^abc,B^	3.39 ± 0.02 ^ab,B^	3.41 ± 0.02 ^c,B^	3.83 ± 0.02 ^bc,A^	3.57 ± 0.00 ^b,B^	3.46 ± 0.02 ^a,A^
B2	t_0_	4.54 ± 0.05 ^a,A^	3.81 ± 0.01 ^c,A^	3.48 ± 0.03 ^d,A^	3.38 ± 0.01 ^d,A^	4.45 ± 0.01 ^a,A^	3.92 ± 0.03 ^a,A^	3.59 ± 0.01 ^c,A^	3.48 ± 0.01 ^ab,A^
	t_1_	4.49 ± 0.07 ^a,A^	3.99 ± 0.00 ^ab,A^	3.70 ± 0.01 ^ab,A^	3.65 ± 0.02 ^ab,A^	4.43 ±0.04 ^ab,A^	4.06 ± 0.03 ^a,A^	3.77 ± 0.01 ^a,A^	3.64 ± 0.05 ^a,A^
	t_2_	4.34 ± 0.02 ^a,A^	3.90 ± 0.08 ^bc,A^	3.58 ± 0.04 ^cd,A^	3.50 ± 0.04 ^c,A^	4.28 ±0.07 ^b,A^	3.73 ± 0.30 ^a,A^	3.67 ± 0.04 ^abc,A^	3.37 ± 0.09 ^b,A^
	t_3_	4.32 ± 0.09 ^a,A^	3.96 ± 0.02 ^b,A^	3.65 ± 0.02 ^bc,A^	3.56 ± 0.02 ^bc,A^	4.35 ± 0.05 ^ab,A^	4.03 ± 0.11 ^a,A^	3.65 ± 0.07 ^abc,A^	3.59 ± 0.01 ^a,A^
	t_4_	4.24 ± 0.11 ^a,A^	3.93 ± 0.02 ^bc,A^	3.60 ± 0.00 ^bc,A^	3.53 ± 0.03 ^c,A^	4.28 ± 0.01 ^b,A^	3.94 ± 0.06 ^a,A^	3.63 ± 0.00 ^bc,A^	3.54 ± 0.00 ^ab,A^
	t_5_	4.34 ± 0.17 ^a,A^	4.09 ± 0.03 ^a,A^	3.79 ± 0.04 ^ab,A^	3.66 ± 0.02 ^ab,A^	4.41 ± 0.03 ^ab,A^	4.06 ± 0.07 ^a,A^	3.75 ± 0.00 ^ab,A^	3.65 ± 0.01 ^a,A^
	t_6_	4.27 ± 0.11 ^a,A^	3.97 ± 0.00 ^ab,A^	3.67 ± 0.01 ^bc,A^	3.59 ± 0.01 ^abc,A^	4.32 ± 0.02 ^ab,A^	4.37 ± 0.64 ^a,A^	3.68 ± 0.01 a^bc,A^	3.51 ± 0.06 ^ab,A^

Values are expressed as means of pickle solution ± standard deviations in each batch (B1, n = 3 replicates; B2, n = 2 replicates). Superscripts indicate statistical significance according to ANOVA and Tukey’s post hoc test. Superscript lowercase letters indicate statistically significant differences between samples within the same column and batch (*p* < 0.05), while superscript uppercase letters indicate statistically significant differences between batches at the same sampling time, within the same column (*p* < 0.05). W1:prototype produced with 0.05% acidity of wine vinegar; W2: prototype produced with 0.2% acidity of wine vinegar; W3: prototype produced with 0.5% acidity of wine vinegar; W4: prototype produced with 0.7% acidity of wine vinegar; A1: prototype produced with 0.05% acidity of apple vinegar; A2: prototype produced with 0.2% acidity of apple vinegar; A3: prototype produced with 0.5% acidity of apple vinegar; A4: prototype produced with 0.7% acidity of apple vinegar.

**Table 5:** Results of TA in laboratory-scale sea fennel pickle prototypes.

Batch	Sampling Time (t, Months)	Prototypes							
		W1	W2	W3	W4	A1	A2	A3	A4
B1	t_0_	0.04 ± 0.01 ^b,A^	0.18 ± 0.00 ^a,B^	0.52 ± 0.01 ^a,A^	0.75 ± 0.02 ^a,A^	0.05 ± 0.01 ^b,A^	0.19 ± 0.01 ^a,A^	0.52 ± 0.01 ^a,A^	0.75 ± 0.01 ^a,A^
	t_6_	0.12 ± 0.02 ^a,A^	0.18 ± 0.00 ^a,B^	0.49 ± 0.01 ^b,B^	0.64 ± 0.01 ^b,B^	0.36 ± 0.02 ^a,A^	0.20 ± 0.01 ^a,A^	0.48 ± 0.00 ^b,A^	0.67 ± 0.00 ^b,B^
B2	t_0_	0.04 ± 0.00 ^b,A^	0.20 ± 0.00 ^b,A^	0.48 ± 0.00 ^b,B^	0.72± 0.01 ^b,A^	0.05 ± 0.00 ^b,A^	0.20 ± 0.00 ^a,A^	0.51 ± 0.01 ^b,A^	0.72 ± 0.01 ^b,B^
	t_6_	0.11 ± 0.01 ^a,A^	0.23 ± 0.02 ^a,A^	0.59 ± 0.02 ^a,A^	0.76 ± 0.00 ^a,A^	0.10 ± 0.00 ^a,B^	0.20 ± 0.00 ^a,A^	0.59 ± 0.02 ^a,A^	0.83 ± 0.01 ^a,A^

Values are expressed as means of pickle solution ± standard deviations of % acetic acid equivalents in each batch (B1, n = 3 replicates; B2, n = 2 replicates). Superscripts indicate statistical significance according to ANOVA and Tukey’s post hoc test. Superscript lowercase letters indicate statistically significant differences between samples within the same column and batch (*p* < 0.05), while superscript uppercase letters indicate statistically significant differences between batches at the same sampling time, within the same column (*p* < 0.05). For legend prototypes see Table 4.

**Table 6:** Results of microbiological counting in laboratory-scale sea fennel pickle prototypes.

Batch	Microbiological Group	Sampling Time (Months)	Prototypes							
B1	Mesophilic lactobacilli (Log CFU mL^−1^)		W1	W2	W3	W4	A1	A2	A3	A4
		t_0_	<1.0	<1.0	<1.0	<1.0	<1.0	<1.0	<1.0	<1.0
		t_1_	<1.0	<1.0	<1.0	<1.0	<1.0	<1.0	<1.0	<1.0
		t_2_	<1.0	<1.0	<1.0	<1.0	<1.0	<1.0	<1.0	<1.0
		t_3_	<1.0	<1.0	<1.0	<1.0	<1.0	<1.0	<1.0	<1.0
		t_4_	<1.0	<1.0	<1.0	<1.0	<1.0	<1.0	<1.0	<1.0
		t_5_	<1.0	<1.0	<1.0	<1.0	<1.0	<1.0	<1.0	<1.0
		t_6_	<1.0	<1.0	<1.0	<1.0	<1.0	<1.0	<1.0	<1.0
	Mesophilic aerobic bacteria (Log CFU mL^−1^)		W1	W2	W3	W4	A1	A2	A3	A4
		t_0_	<1.0 ^b,A^	<1.0	<1.0	<1.0	<1.0 ^c,A^	<1.0	<1.0	<1.0
		t_1_	<1.0 ^b,A^	<1.0	<1.0	<1.0	<1.0 ^c,A^	<1.0	<1.0	<1.0
		t_2_	2.5 ± 2.2 ^ab,A^	<1.0	<1.0	<1.0	<1.0 ^c,A^	<1.0	<1.0	<1.0
		t_3_	2.5 ± 2.1 ^ab,A^	<1.0	<1.0	<1.0	<1.0 ^c,A^	<1.0	<1.0	<1.0
		t_4_	2.9 ± 2.5 ^ab,A^	<1.0	<1.0	<1.0	<1.0 ^c,A^	<1.0	<1.0	<1.0
		t_5_	4.0 ± 0.2 ^ab,A^	<1.0	<1.0	<1.0	2.7 ± 0.1 ^b,A^	<1.0	<1.0	<1.0
		t_6_	4.7 ± 0.1 ^a,A^	<1.0	<1.0	<1.0	4.6 ± 0.1 ^a,A^	<1.0	<1.0	<1.0
	Yeasts (Log CFU mL^−1^)		W1	W2	W3	W4	A1	A2	A3	A4
		t_0_	<1.0 ^b,A^	<1.0	<1.0	<1.0	<1.0 ^a,A^	<1.0	<1.0	<1.0
		t_1_	<1.0 ^b,A^	<1.0	<1.0	<1.0	<1.0 ^a,A^	<1.0	<1.0	<1.0
		t_2_	<1.0 ^b,A^	<1.0	<1.0	<1.0	<1.0 ^a,A^	<1.0	<1.0	<1.0
		t_3_	3.5 ± 0.2 ^a,A^	<1.0	<1.0	<1.0	1.2 ± 2.5 ^a,A^	<1.0	<1.0	<1.0
		t_4_	3.6 ± 0.2 ^a,A^	<1.0	<1.0	<1.0	1.7 ± 2.4 ^a,A^	<1.0	<1.0	<1.0
		t_5_	3.5 ± 0.1 ^a,A^	<1.0	<1.0	<1.0	3.5 ± 0.0 ^a,A^	<1.0	<1.0	<1.0
		t_6_	3.6 ± 0.1 ^a,A^	<1.0	<1.0	<1.0	3.6 ± 0.1 ^a,A^	<1.0	<1.0	<1.0
	Molds (Log CFU mL^−1^)		W1	W2	W3	W4	A1	A2	A3	A4
		t_0_	<1.0 ^c^	<1.0	<1.0	<1.0	<1.0	<1.0	<1.0	<1.0
		t_1_	<1.0 ^c^	<1.0	<1.0	<1.0	<1.0	<1.0	<1.0	<1.0
		t_2_	1.65 ± 2.85 ^b^	<1.0	<1.0	<1.0	<1.0	<1.0	<1.0	<1.0
		t_3_	3.24 ± 2.81 ^a^	<1.0	<1.0	<1.0	<1.0	<1.0	<1.0	<1.0
		t_4_	<1.0 ^c^	<1.0	<1.0	<1.0	<1.0	<1.0	<1.0	<1.0
		t_5_	<1.0 ^c^	<1.0	<1.0	<1.0	<1.0	<1.0	<1.0	<1.0
		t_6_	<1.0 ^c^	<1.0	<1.0	<1.0	<1.0	<1.0	<1.0	<1.0
	Enterobacteriaceae (Log CFU mL^−1^)		W1	W2	W3	W4	A1	A2	A3	A4
		t_0_	<1.0	<1.0	<1.0	<1.0	<1.0	<1.0	<1.0	<1.0
		t_1_	<1.0	<1.0	<1.0	<1.0	<1.0	<1.0	<1.0	<1.0
		t_2_	<1.0	<1.0	<1.0	<1.0	<1.0	<1.0	<1.0	<1.0
		t_3_	<1.0	<1.0	<1.0	<1.0	<1.0	<1.0	<1.0	<1.0
		t_4_	<1.0	<1.0	<1.0	<1.0	<1.0	<1.0	<1.0	<1.0
		t_5_	<1.0	<1.0	<1.0	<1.0	<1.0	<1.0	<1.0	<1.0
		t_6_	<1.0	<1.0	<1.0	<1.0	<1.0	<1.0	<1.0	<1.0
B2	Mesophilic lactobacilli (Log CFU mL^−1^)		W1	W2	W3	W4	A1	A2	A3	A4
		t_0_	<1.0	<1.0	<1.0	<1.0	<1.0	<1.0 ^a^	<1.0	<1.0
		t_1_	<1.0	<1.0	<1.0	<1.0	<1.0	2.1 ± 3.0 ^a^	<1.0	<1.0
		t_2_	<1.0	<1.0	<1.0	<1.0	<1.0	1.6 ± 2.2 ^a^	<1.0	<1.0
		t_3_	<1.0	<1.0	<1.0	<1.0	<1.0	1.5 ± 2.2 ^a^	<1.0	<1.0
		t_4_	<1.0	<1.0	<1.0	<1.0	<1.0	1.5 ± 2.2 ^a^	<1.0	<1.0
		t_5_	<1.0	<1.0	<1.0	<1.0	<1.0	<1.0 ^a^	<1.0	<1.0
		t_6_	<1.0	<1.0	<1.0	<1.0	<1.0	<1.0 ^a^	<1.0	<1.0
	Mesophilic aerobic bacteria (Log CFU mL^−1^)		W1	W2	W3	W4	A1	A2	A3	A4
		t_0_	<1.0 ^a,A^	<1.0	<1.0	<1.0	<1.0 ^c,A^	<1.0 ^a^	<1.0	<1.0
		t_1_	<1.0 ^a,A^	<1.0	<1.0	<1.0	<1.0 ^c,A^	2.1 ± 3.03 ^a^	<1.0	<1.0
		t_2_	<1.0 ^a,A^	<1.0	<1.0	<1.0	<1.0 ^c,A^	1.5 ± 2.15 ^a^	<1.0	<1.0
		t_3_	<1.0 ^a,A^	<1.0	<1.0	<1.0	<1.0 ^c,A^	1.5 ± 2.18 ^a^	<1.0	<1.0
		t_4_	<1.0 ^a,A^	<1.0	<1.0	<1.0	<1.0 ^c,A^	1.6 ± 2.19 ^a^	<1.0	<1.0
		t_5_	1.3 ± 1.8 ^a,A^	<1.0	<1.0	<1.0	2.7 ± 0.1 ^b,A^	1.3 ± 1.76 ^a^	<1.0	<1.0
		t_6_	<1.0 ^a,B^	<1.0	<1.0	<1.0	4.6 ± 0.1 ^a,A^	<1.0 ^a^	<1.0	<1.0
	Yeasts (Log CFU mL^−1^)		W1	W2	W3	W4	A1	A2	A3	A4
		t_0_	<1.0 ^a,A^	<1.0 ^a^	<1.0	<1.0	<1.0 ^a,A^	<1.0 ^a^	<1.0	<1.0
		t_1_	1.4 ± 1.9 ^a,A^	<1.0 ^a^	<1.0	<1.0	1.9 ± 2.64 ^a,A^	<1.0 ^a^	<1.0	<1.0
		t_2_	1.9 ± 2.6 ^a,A^	<1.0 ^a^	<1.0	<1.0	1.2 ± 1.67 ^a,A^	<1.0 ^a^	<1.0	<1.0
		t_3_	1.3 ± 1.8 ^a,A^	<1.0 ^a^	<1.0	<1.0	1.1 ± 1.41 ^a,A^	<1.0 ^a^	<1.0	<1.0
		t_4_	1.2 ± 1.8 ^a,A^	<1.0 ^a^	<1.0	<1.0	2.4 ± 3.42 ^a,A^	<1.0 ^a^	<1.0	<1.0
		t_5_	1.2 ± 1.7 ^a,A^	1.4 ± 2.0 ^a^	<1.0	<1.0	1.5 ± 2.16 ^a,A^	<1.0 ^a^	<1.0	<1.0
		t_6_	<1.0 ^a,B^	<1.0 ^a^	<1.0	<1.0	<1.0 ^a,A^	1.5 ± 2.1 ^a^	<1.0	<1.0
	Molds (Log CFU mL^−1^)		W1	W2	W3	W4	A1	A2	A3	A4
		t_0_	<1.0	<1.0	<1.0	<1.0	<1.0	<1.0	<1.0	<1.0
		t_1_	<1.0	<1.0	<1.0	<1.0	<1.0	<1.0	<1.0	<1.0
		t_2_	<1.0	<1.0	<1.0	<1.0	<1.0	<1.0	<1.0	<1.0
		t_3_	<1.0	<1.0	<1.0	<1.0	<1.0	<1.0	<1.0	<1.0
		t_4_	<1.0	<1.0	<1.0	<1.0	<1.0	<1.0	<1.0	<1.0
		t_5_	<1.0	<1.0	<1.0	<1.0	<1.0	<1.0	<1.0	<1.0
		t_6_	<1.0	<1.0	<1.0	<1.0	<1.0	<1.0	<1.0	<1.0
	Enterobacteriaceae (Log CFU mL^−1^)		W1	W2	W3	W4	A1	A2	A3	A4
		t_0_	<1.0	<1.0	<1.0	<1.0	<1.0	<1.0	<1.0	<1.0
		t_1_	<1.0	<1.0	<1.0	<1.0	<1.0	<1.0	<1.0	<1.0
		t_2_	<1.0	<1.0	<1.0	<1.0	<1.0	<1.0	<1.0	<1.0
		t_3_	<1.0	<1.0	<1.0	<1.0	<1.0	<1.0	<1.0	<1.0
		t_4_	<1.0	<1.0	<1.0	<1.0	<1.0	<1.0	<1.0	<1.0
		t_5_	<1.0	<1.0	<1.0	<1.0	<1.0	<1.0	<1.0	<1.0
		t_6_	<1.0	<1.0	<1.0	<1.0	<1.0	<1.0	<1.0	<1.0

Values are expressed as mean Log CFU mL^−1^ of pickle solution ± standard deviations in each batch (B1, n = 3 replicates; B2, n = 2 replicates). Superscripts indicate statistical significance according to ANOVA and Tukey’s post hoc test in yeasts and mesophilic aerobic bacteria groups. For each microbial group, Superscript lowercase letters indicate statistically significant differences between samples within the same column for each microbial group and batch (*p* < 0.05), while superscript uppercase letters indicate statistically significant differences between batches at the same sampling time, within the same column and microbial group (*p* < 0.05). For legend prototypes see Table 4.

**Table 7:** Colorimetric profile of leaves in laboratory-scale sea fennel pickle prototypes.

Batch	Prototype	Sampling Time (t, Months)	Color Parameter				
			L*	a*	b*	h°	C
B1	W2						
		t_0_	35.92 ± 2.20 ^a,A^	−4.05 ± 0.04 ^a,A^	14.89 ± 1.10 ^a,A^	103.26 ± 0.72 ^a,A^	17.70 ± 1.08 ^a,A^
		t_3_	34.79 ± 1.47 ^a,B^	−3.03 ± 1.07 ^ab,A^	11.92 ± 3.21 ^b,A^	104.08 ± 1.95 ^a,A^	12.30 ± 3.36 ^b,A^
		t_6_	37.24 ± 1.66 ^a,A^	−2.19 ± 0.43 ^b,B^	10.17 ± 1.17 ^b,B^	102.18 ± 2.36 ^a,A^	10.41 ± 1.18 ^b,B^
	W3						
		t_0_	36.34 ± 3.03 ^a,A^	−3.27 ± 1.46 ^a,A^	17.79 ± 3.36 ^a,A^	100.08 ± 2.64 ^a,A^	18.10 ± 3.57 ^a,A^
		t_3_	34.35 ± 0.81 ^a,B^	−2.04 ± 0.25 ^a,B^	8.23 ± 0.46 ^a,B^	103.99 ± 2.38 ^a,A^	8.49 ± 0.39 ^b,B^
		t_6_	37.02 ± 3.06 ^a,A^	−2.23 ± 0.50 ^a,A^	9.10 ± 1.06 ^b,B^	103.67 ± 1.61 ^a,A^	9.37 ± 1.15 ^b,B^
	W4						
		t_0_	35.43 ± 1.90 ^a,A^	−3.14 ± 0.96 ^a,A^	15.58 ± 3.59 ^a,A^	101.27 ± 1.61 ^a,A^	15.90 ± 3.69 ^a,A^
		t_3_	36.20 ± 1.21 ^a,A^	−2.37 ± 0.89 ^a,A^	9.59 ± 2.50 ^a,A^	103.58 ± 2.34 ^a,A^	9.88 ± 2.63 ^a,A^
		t_6_	33.86 ± 1.50 ^b,A^	−2.75 ± 0.84 ^a,A^	8.91 ± 2.04 ^b,A^	106.92 ± 2.06 ^a,A^	9.33 ± 2.19 ^a,A^
	A2						
		t_0_	33.51 ± 1.31 ^b,B^	−2.77 ± 0.13 ^a,B^	13.31 ± 1.88 ^a,B^	101.95 ± 2.30 ^a,A^	13.60 ± 1.81 ^a,B^
		t_3_	34.27 ± 2.12 ^b,A^	−2.67 ± 0.90 ^a,A^	12.30 ± 3.50 ^a,A^	102.15 ± 0.74 ^a,A^	12.59 ± 3.61 ^a,A^
		t_6_	40.76 ± 1.96 ^a,B^	−2.89 ± 1.59 ^a,A^	14.34 ± 1.42 ^a,A^	101.12 ± 5.02 ^a,A^	14.65 ± 1.70 ^a,A^
	A3						
		t_0_	33.29 ± 1.92 ^a,A^	−2.36 ± 1.45 ^a,A^	13.66 ± 2.32 ^a,A^	99.27 ± 4.77 ^a,A^	13.89 ± 2.51 ^a,A^
		t_3_	33.31 ± 0.38 ^a,B^	−1.82 ± 0.60 ^a,A^	7.85 ± 1.27 ^a,B^	102.77 ± 2.45 ^a,A^	8.06 ± 1.36 ^b,B^
		t_6_	35.16 ± 2.06 ^a,A^	−2.65 ± 0.17 ^a,B^	9.26 ± 0.85 ^b,B^	105.99 ± 0.55 ^a,A^	9.63 ± 0.86 ^ab,B^
	A4						
		t_0_	33.72 ± 1.48 ^a,A^	−3.52 ± 0.88 ^a,B^	14.95 ± 2.67 ^a,A^	103.16 ± 0.99 ^a,A^	15.36 ± 2.79 ^a,A^
		t_3_	32.33 ± 0.88 ^a,B^	−1.83 ± 0.49 ^b,A^	8.89 ± 2.00 ^b,B^	101.78 ± 2.75 ^a,A^	9.08 ± 2.00 ^b,B^
		t_6_	33.33 ± 0.79 ^a,A^	−1.60 ± 0.09 ^b,A^	7.32 ± 0.38 ^b,B^	102.32 ± 0.12 ^a,A^	7.49 ± 0.39 ^b,B^
B2	W2						
		t_0_	35.08 ± 0.36 ^b,A^	−5.48 ± 1.58 ^a,A^	19.29 ± 2.21 ^a,A^	105.70 ± 2.64 ^a,A^	20.06 ± 2.56 ^a,A^
		t_3_	44.12 ± 1.70 ^a,A^	−3.40 ± 0.50 ^a,A^	17.71 ± 1.22 ^a,A^	100.85 ± 0.83 ^a,A^	18.03 ± 1.29 ^a,A^
		t_6_	38.17 ± 0.55 ^b,A^	−4.56 ± 0.02 ^a,A^	15.79 ± 0.04 ^a,A^	106.10 ± 0.11 ^a,A^	16.43 ± 0.03 ^b,A^
	W3						
		t_0_	35.80 ± 0.96 ^b,A^	−5.55 ± 0.05 ^a,A^	17.20 ± 0.59 ^a,A^	107.88 ± 0.73 ^a,A^	18.07 ± 0.55 ^a,A^
		t_3_	38.91 ± 0.07 ^a,A^	−4.28 ± 0.01 ^a,A^	17.56 ± 3.13 ^a,A^	103.90 ± 2.43 ^a,A^	18.08 ± 3.04 ^a,A^
		t_6_	36.03 ± 1.45 ^ab,A^	−2.95 ± 1.10 ^a,A^	14.06 ± 1.94 ^a,A^	101.63 ± 2.72 ^a,A^	14.37 ± 2.13 ^b,A^
	W4						
		t_0_	38.67 ± 2.02 ^a,A^	−4.90 ± 0.10 ^a,A^	19.72 ± 1.19 ^a,A^	103.98 ± 0.53 ^a,A^	20.32 ± 1.18 ^a,A^
		t_3_	37.37 ± 1.88 ^a,A^	−3.40 ± 0.13 ^b,A^	14.34 ± 0.04 ^b,A^	103.34 ± 0.47 ^a,A^	14.34 ± 0.04 ^b,A^
		t_6_	35.12 ± 2.91 ^a,A^	−3.20 ± 0.59 ^b,A^	13.46 ± 1.12 ^b,A^	103.50 ± 3.44 ^a,A^	13.84 ± 0.95 ^b,A^
	A2						
		t_0_	38.26 ± 1.50 ^a,A^	−5.71 ± 0.14 ^a,A^	20.44± 0.24 ^a,A^	105.62 ±0.55 ^a,A^	21.22 ± 0.19 ^a,A^
		t_3_	40.07 ± 2.56 ^a,A^	−3.30 ± 0.50 ^b,A^	12.98± 1.09 ^b,A^	104.35 ± 3.23 ^a,A^	13.40 ± 0.93 ^b,A^
		t_6_	32.75 ± 0.94 ^a,A^	−3.51 ± 0.66 ^b,A^	14.90 ± 2.82 ^ab,A^	103.25 ± 0.01 ^a,A^	15.30 ± 2.90 ^ab,A^
	A3						
		t_0_	32.24 ± 0.63 ^a,A^	−3.65 ± 0.29 ^a,A^	14.70 ± 0.44 ^a,A^	103.95 ± 1.47 ^a,A^	15.15 ± 0.36 ^a,A^
		t_3_	37.66 ± 1.06 ^a,A^	−3.41 ± 0.94 ^a,A^	14.13 ± 1.33 ^a,A^	103.45 ± 2.40 ^a,A^	14.54 ± 1.51 ^a,A^
		t_6_	35.79 ± 4.38 ^a,A^	−4.10 ± 0.12 ^a,A^	15.35 ± 1.18 ^a,A^	105.00 ± 1.54 ^a,A^	15.89 ± 1.11 ^a,A^
	A4						
		t_0_	35.82 ± 2.29 ^a,A^	−4.43 ± 1.38 ^a,A^	16.67 ± 3.28 ^a,A^	104.70 ± 1.68 ^a,A^	17.25 ± 2.79 ^a,A^
		t_3_	37.12 ± 0.07 ^a,A^	−3.44 ± 0.30 ^a,A^	14.37 ± 0.27 ^a,A^	103.46 ± 1.38 ^a,A^	14.78 ± 0.19 ^a,A^
		t_6_	36.27 ± 2.13 ^a,A^	−3.22 ± 0.98 ^a,A^	13.42 ± 2.10 ^a,A^	103.33 ± 1.96 ^a,A^	13.81 ± 2.27 ^a,A^

CIELAB color parameters: L, lightness; a*, redness–greenness; b*, yellowness–blueness; h°, hue angle; C, chroma. Results are expressed as means of pickled sea fennel leaves samples ± standard deviation in each batch (B1, n = 3 replicates; B2, n = 2 replicates). For each parameter and prototype within the same batch, values labeled with different small letters in the same column are significantly different (*p* < 0.05), whereas, for each parameter, prototype and sampling time, values labeled with different capital letters in the same column are significantly different between the two batches (*p* < 0.05). For legend prototypes see Table 4.

**Table 8:** Results of salt measurement in laboratory-scale sea fennel pickle prototypes.

Sampling Time (t, Months)	Sample	B1	B2
t_0_			
	W2	2.62 ± 0.12 ^a,A^	2.45 ± 0.57 ^a,A^
	W3	2.25 ± 0.28 ^a,A^	2.15 ± 0.21 ^a,A^
	W4	2.43 ± 0.28 ^a,A^	2.23 ± 0.28 ^a,A^
	A2	2.05 ± 0.26 ^a,A^	2.10 ± 0.00 ^a,A^
	A3	2.68 ± 0.06 ^a,A^	2.33 ± 0.28 ^a,A^
	A4	2.61 ± 0.32 ^a,A^	2.33 ± 0.40 ^a,A^
t_6_			
	W2	1.05 ± 0.00 ^b,B^	2.00 ± 0.00 ^a,A^
	W3	1.65 ± 0.13 ^b,A^	1.20 ± 0.07 ^b,B^
	W4	1.48 ± 0.08 ^b,A^	1.45 ± 0.07 ^b,A^
	A2	1.32 ± 0.06 ^b,A^	1.20 ± 0.00 ^b,A^
	A3	1.70 ± 0.28 ^b,A^	1.28 ± 0.11 ^b,A^
	A4	1.66 ± 0.21 ^b,A^	1.27 ±0.18 ^b,A^

Values are expressed as means of pickled sea fennel leaves and stems samples ± standard deviations of % NaCl in each batch (B1, n = 3 replicates; B2, n = 2 replicates). Superscripts indicate statistical significance according to ANOVA and Tukey’s post hoc test. Superscript lowercase letters indicate statistically significant differences between samples within the same column, at each sampling time (*p* < 0.05), while superscript uppercase letters indicate statistically significant differences between batches, for each prototype within the same row (*p* < 0.05). For legend prototypes see Table 4.

**Table 9:** Groups of volatile organic compounds (VOCs) detected by HS-SPME-GC-MS in leaves and stems of laboratory-scale sea fennel pickle prototypes in batch 1 (B1) at month 0 (T0) and after 6 months of storage (T6), treated with apple or wine vinegars at acidity levels of 0.2% and 0.7%.

No.	Compounds	RI (est)	RI(lit)	A2		A4		W2		W4	
				t_0_	t_6_	t_0_	t_6_	t_0_	t_6_	t_0_	t_6_
Monoterpenes											
1	Limonene	1186	1169	48.49 ± 1.87	18.19 ± 0.81	42.96 ± 1.63	25.68 ± 1.44	50.09 ± 1.03	24.41 ± 1.88	41.41 ± 1.40	24.81 ± 1.52
2	γ-Terpinene	1234	1250	16.49 ± 0.55	n.d.	16.50 ± 0.52	n.d.	10.71 ± 0.45	n.d.	17.34 ± 1.05	n.d.
3	p-Cymene	1262	1288	33.06 ± 1.25	61.51 ± 2.18	29.38 ± 1.79	58.97 ± 1.07	34.62 ± 1.45	54.25 ± 2.13	32.83 ± 1.34	51.11 ± 1.90
Aromatic Monoterpenes											
4	p-Cymenene	1430	1414	n.d.	0.44 ± 0.11	n.d.	0.29 ± 0.04	n.d.	0.40 ± 0.07	n.d.	0.18 ± 0.08
Aldehydes											
5	Octanal	1285	1288	n.d.	1.68 ± 0.20	0.39 ± 0.17	1.18 ± 0.19	n.d.	2.46 ± 0.43	n.d.	1.64 ± 0.19
6	2-heptenal	1317	1334	n.d.	n.d.	n.d.	0.38 ± 0.03	n.d.	n.d.	n.d.	1.39 ± 0.07
7	2-octenal	1423	1412	n.d.	n.d.	n.d.	n.d.	n.d.	n.d.	n.d.	0.30 ± 0.08
Carboxylic Acid											
8	Acetic acid	1460	1439	0.90 ± 0.12	3.36 ± 0.32	7.37 ± 0.40	7.10 ± 0.65	2.75 ± 0.59	2.84 ± 0.47	5.29 ± 1.07	8.40 ± 0.85
Phenolic Derivatives											
9	Thymol methyl ether	1576	1595	0.21 ± 0.04	0.45 ± 0.07	n.d.	0.22 ± 0.04	n.d.	n.d.	0.29 ± 0.03	0.25 ± 0.11
10	Thymol methyl ether isomer	1591	1595	n.d.	n.d.	n.d.	n.d.	n.d.	n.d.	0.21 ± 0.04	0.23 ± 0.09
Sesquiterpene											
11	α-Curcumene	1761	1782	n.d.	n.d.	n.d.	n.d.	n.d.	n.d.	0.28 ± 0.04	1.62 ± 0.07
Terpenoids											
12	Terpinen-4-ol	1595	1602	0.80 ± 0.10	n.d.	1.61 ± 0.26	0.39 ± 0.05	1.48 ± 0.20	1.13 ± 0.56	1.46 ± 0.29	0.80 ± 0.08
13	α-Terpineol	1693	1683	n.d.	0.66 ± 0.09	0.75 ± 0.05	0.83 ± 0.16	n.d.	1.61 ± 0.40	0.43 ± 0.09	2.43 ± 0.55
14	Carvone	1720	1728	n.d.	6.11 ± 0.13	0.65 ± 0.05	2.56 ± 0.31	n.d.	6.25 ± 0.90	0.15 ± 0.04	4.02 ± 0.88
15	Isocarveol	1793	1797	n.d.	2.97 ± 0.07	n.d.	0.87 ± 0.09	n.d.	3.17 ± 0.73	n.d.	n.d.
16	p-Mentha-1(7),8-dien-2-ol	1885	1877	n.d.	1.98 ± 0.05	n.d.	0.53 ± 0.06	n.d.	1.56 ± 0.31	0.18 ± 0.03	0.72 ± 0.18
	Total			99.95	97.36	99.61	99.00	99.65	98.07	99.88	97.91

Values are expressed as relative percentage of total peak area (average ± standard deviation, n = 2), n.d.: not detected. Experimental retention indices (RIest) and literature retention indices (RIlit), obtained using GC analysis with a polyethylene glycol-coated capillary column, are also reported. t_0_: sampling time at the beginning of storage (Month 0); t_6_: sampling time at the end of storage (Month 6). For legend, see Table 4.

**Table 10:** Groups of volatile organic compounds (VOCs) detected by HS-SPME-GC-MS in leaves and stems of laboratory-scale sea fennel pickle prototypes in batch 2 at month 0 (T0) and after 6 months of storage (T6), treated with apple or wine vinegars at acidity levels of 0.2% and 0.7%.

No.	Compounds	RI(est)	RI(lit)	A2		A4		W2		W4	
				t_0_	t_6_	t_0_	t_6_	t_0_	t_6_	t_0_	t_6_
Monoterpenes											
1	β-Myrcene	1156	1157	0.51 ± 0.08	n.d.	n.d.	n.d.	0.45 ± 0.11	n.d.	0.20 ± 0.08	0.19 ± 0.10
2	α-Terpinene	1162	1167	0.36 ± 0.14	n.d.	n.d.	n.d.	n.d.	n.d.	n.d.	n.d.
3	Limonene	1186	1169	27.54 ± 1.36	19.33 ± 1.49	16.51 ± 1.39	16.98 ± 0.72	27.67 ± 1.55	31.81 ± 1.18	21.93 ±1.91	32.96 ± 1.49
4	β-Phellandrene	1191	1188	1.26 ± 0.11	0.51 ± 0.18	0.91 ± 0.11	0.56 ± 0.08	0.91 ± 0.14	0.62 ± 0.12	0.84 ± 0.07	0.71 ± 0.05
5	γ-Terpinene	1234	1250	28.91 ± 1.23	2.15 ± 0.50	10.75 ± 1.09	3.22 ± 0.53	12.88 ± 1.01	2.87 ± 0.75	18.48 ±1.67	5.99 ± 0.97
6	α-Ocimene	1249	1250	0.04 ± 0.04	n.d.	n.d.	n.d.	n.d.	n.d.	n.d.	n.d.
7	p-Cymene	1262	1288	12.80 ± 1.18	42.79 ± 1.18	24.95 ± 1.29	35.32 ±1.03	28.14 ± 1.05	39.46 ± 1.10	13.04 ± 1.01	32.24 ± 1.20
8	Terpinolene	1270	1283	0.65 ± 0.09	n.d.	n.d.	n.d.	n.d.	n.d.	n.d.	0.12 ± 0.03
Aromatic Monoterpenes											
9	p-Cymenene	1430	1414	n.d.	0.27 ± 0.07	n.d.	0.46 ± 0.04	n.d.	0.16 ± 0.06	n.d.	0.20 ± 0.02
Carboxylic Acid											
10	Acetic acid	1460	1439	0.19 ± 0.02	0.59 ± 0.08	4.15 ± 0.14	2.32 ± 0.32	0.39 ± 0.07	0.47 ± 0.02	2.54 ± 0.11	1.75 ± 0.07
Sesquiterpene											
11	trans-α-Bergamotene	1571	1574	0.16 ± 0.01	n.d.	n.d.	n.d.	0.05 ± 0.05	n.d.	0.32 ± 0.02	0.15 ± 0.02
12	β-Bisabolene	1713	1736	0.15 ± 0.01	n.d.	n.d.	n.d.	n.d.	n.d.	0.25 ± 0.05	0.07 ± 0.07
13	(-)-β-Sesquiphellandrene	1755	1754	0.38 ± 0.07	n.d.	n.d.	n.d.	0.04 ± 0.04	0.00 ± 0.00	0.54 ± 0.19	0.16 ± 0.04
14	α-Curcumene	1761	1782	n.d.	n.d.	n.d.	n.d.	0.10 ± 0.01	0.05 ± 0.05	0.09 ± 0.09	0.05 ± 0.05
Phenolic Derivatives											
15	Thymol methyl ether	1576	1595	0.25 ± 0.03	0.37 ± 0.08	0.18 ± 0.09	0.38 ± 0.05	0.21 ± 0.05	0.16 ± 0.05	0.19 ± 0.06	0.18 ± 0.06
16	Thymol methyl ether isomer	1591	1595	19.47 ± 0.69	27.46 ± 1.96	27.20 ± 1.90	30.15 ± 1.13	21.49 ± 1.41	15.40 ± 1.65	23.57 ± 1.88	17.36 ± 1.46
17	Dill apiol	2348	2351	2.52 ± 0.41	1.11 ± 0.10	4.65 ± 0.45	1.62 ± 0.20	1.59 ± 0.44	1.49 ± 0.16	3.06 ± 0.59	2.26 ± 0.09
Terpenoids											
18	Terpinen-4-ol	1595	1602	4.57 ± 0.91	3.76 ± 0.63	10.27 ± 0.83	6.30 ± 0.36	5.10 ± 0.96	6.86 ± 0.59	14.41 ± 0.49	4.66 ± 0.33
19	α-Terpineol	1693	1683	n.d.	0.12 ± 0.03	0.28 ± 0.06	0.31 ± 0.11	0.11 ± 0.03	0.20 ± 0.09	0.20 ± 0.08	0.24 ± 0.05
20	Carvone	1720	1728	n.d.	0.38 ± 0.04	n.d.	0.70 ± 0.14	0.03 ± 0.03	0.13 ± 0.07	n.d.	0.11 ± 0.03
21	Isocarveol	1793	1797	n.d.	0.49 ± 0.06	n.d.	0.68 ± 0.12	n.d.	0.15 ± 0.08	n.d.	0.13 ± 0.03
22	p-Mentha-1(7),8-dien-2-ol	1885	1877	n.d.	0.35 ± 0.06	n.d.	0.46 ± 0.13	0.03 ± 0.03	0.08 ± 0.08	n.d.	0.06 ± 0.06
Aldehydes/Ketones											
23	6,8-Nonadien-2-one, 6-methyl-5-(1-methylethylidene)-	1705	1767	n.d.	n.d.	n.d.	n.d.	0.66 ± 0.20	n.d.	0.03 ± 0.03	n.d.
	Total			99.76	99.69	99.83	99.44	99 84	99.92	99.69	99.59

Values are expressed as relative percentage of total peak area (average ± standard deviation, n = 2), n.d.: not detected. Experimental retention indices (RIest) and literature retention indices (RIlit), obtained using GC analysis with a polyethylene glycol-coated capillary column, are also reported. t_0_: sampling time at the beginning of storage (Month 0); t_6_: sampling time at the end of storage (Month 6). For legend, see Table 4.

## Data Availability

The original contributions presented in this study are included in the article. Further inquiries can be directed to the corresponding author.
